# Researcher, research thyself? Mapping the landscape of canine health and welfare research funding provided by UK not-for-profit organisations from 2012–2022

**DOI:** 10.1371/journal.pone.0303498

**Published:** 2024-05-23

**Authors:** Alison M. Skipper, Rowena M. A. Packer, Dan G. O’Neill

**Affiliations:** 1 Department of Pathobiology and Population Sciences, The Royal Veterinary College, Hawkshead Lane, North Mymms, Hatfield, Hertfordshire, United Kingdom; 2 Department of Clinical Science and Services, The Royal Veterinary College, Hawkshead Lane, North Mymms, Hatfield, Hertfordshire, United Kingdom; Kerman University of Medical Sciences, ISLAMIC REPUBLIC OF IRAN

## Abstract

**Background:**

Research into canine health and welfare is supported by Government, charitable and private UK funding organisations. However, there is no current overall visibility or coordination of these funding activities, potentially compromising optimal distribution of limited resources. This study aimed to survey UK canine health and welfare funding by not-for-profit funders between 2012 and 2022, providing a novel baseline analysis to inform future sector stakeholder priorities.

**Results:**

Funding data were collected from 10 wide-scope funders (UK Government funding councils and medical charities), 18 animal-directed funders (organisations specifically concerned with animal health and welfare) and 81 breed community groups. These 109 UK funders together provided traceable canine-relevant funding of £57.8 million during the surveyed period, comprising 684 individual grant awards supporting over 500 separate research projects. Wide-scope funders contributed £41.2 million (71.2% of total funding); animal-directed organisations, £16.3 million (28.1% of total funding); and breed-specific groups, £370K (0.6% of total funding). Individual grants ranged from £2.3 million to £300. Funding patterns varied between sectors. Animal-directed funders provided £14.7 million of canine-relevant research funding that foregrounded the dog, 73% of all such funding; wide-scope funders provided £17.5 million of canine-relevant One Health research funding, 97% of all such funding. Customised metrics developed for this study assessed the ‘benefit to the dog’ and ‘pathway to impact’ of individual research projects. Overall, studies supported by animal-directed funders achieved significantly higher ‘benefit to the dog’ scores (Mann-Whitney U = 45235, *p*<0.001) and ‘pathway to impact’ scores (Mann-Whitney U = 43506.5, *p*<0.001) than those supported by wide-scope funders.

**Conclusion:**

The landscape of UK not-for-profit funding of canine health and welfare research is complex, with considerable variation between providers. Although wide-scope funders provide the majority of overall canine-relevant research funding, animal-directed funders provide the majority of canine-focused funding and support research with greater direct impact on canine welfare. Visibility of past funding patterns will enable stakeholders in this sector to make more informed decisions about future research.

**Definitions:**

To increase clarity, certain words and phrases are used in specific ways within the context of this paper.

**Animal-directed funders**—Charities and other funding organisations whose remit primarily concerns animals or veterinary work

**Canine-focused research**—Investigations where the primary purpose is to advance understandings of canine health and/or welfare

**Canine-relevant research**—All research that is framed as advancing understandings of canine health and/or welfare as a primary or subsidiary purpose

**Institution—**Refers to universities and other centres where research is carried out

**Organisation**—Refers to funding bodies, including research councils, charities and other groups

**Research grant**—A single funding event originating from one or more funders

**Research project**—A cohesive piece of research concerning a particular topic; may involve multiple researchers and/or multiple research grants, in series or in parallel

**Wide-scope funders**—Large organisations whose remit does not primarily concern animals, i.e. (in this dataset) UKRI councils and the Wellcome Trust

## 1. Introduction

First domesticated over 30,000 years ago, the dog is our longest-established companion animal and (with the cat) remains the most numerous mammalian companion species. In 2023, the UK domestic dog population was estimated at 11 million [1,2]. As a species, the domestic dog has literally been shaped by humanity in both its physical form and behaviour [3]. Dogs occupy multiple roles in modern societies, such as domestic companion, agricultural worker, service animal, laboratory model and free-living commensal [4]. Moreover, dogs’ bodies and minds have long been the subjects of academic research, because of the similarity of their biological processes to our own, the impact of zoonotic disease on human health and their significance to humans as companion animals, which drives societal concern for their welfare [5]. Such research is enormously variable, ranging from laboratory investigations where dogs serve as biological resources to social science studies that explore the human-animal bond [6]. Consequently, canine research takes place in multiple locations, across multiple disciplines, and is funded in multiple ways, ranging from competitive formal grant awards to ad-hoc informal support from owner communities. Yet the extent and nature of these research activities in the UK has never previously been systematically documented, apart from the annual numerical Home Office data that records the dogs used in scientific procedures, one small area of the wider canine science landscape [7].

Because canine-relevant research investigations are so disparate and fragmented, it is challenging to identify all activity in this sector. However, a significant quantity of research into canine health and welfare is formally financed through research grants, which may be awarded by major public bodies such as UKRI councils (UK Research and Innovation; www.UKRI.org), by large charities such as the Wellcome Trust (a major independent funder of medical research; www.wellcome.org), or through specialised charities and other organisations that are concerned with animals in general, dogs in particular, or even individual breeds. Each of these funders generally evaluates research proposals separately, using their own organisational processes. For example, some funders issue time-limited calls for proposals that address specific research themes, while others offer ongoing open funding calls. Although all funders use grant review committees to assess research proposals, some operate formalised scoring systems and refer all proposals for specialist review, while others assess proposals more informally, and refer some proposals for specialist review on an occasional ad-hoc basis. Consequently, funding decisions are usually produced in relative isolation, apart from a small proportion of jointly commissioned or funded projects. However, these current funding processes can lead to inefficiencies both for funders, who duplicate effort and expertise in these evaluations, and for researchers, who often must tailor multiple applications to multiple organisations to maximise their chances of successfully securing funding for each research proposal. Moreover, as there is currently no overarching visibility of the wider funding landscape, important areas of research may be inadvertently neglected while others attract multiple overlapping investigations, consuming limited resources that could have a greater positive impact for dogs if deployed elsewhere. These issues potentially reduce the overall effectiveness of UK-funded research into canine health and welfare, both for advancing the aims of human and organisational stakeholders in this sector and for benefitting the dogs themselves.

### 1.1 Aims

Recognising the many difficulties around optimising funding and outcomes for canine research, in 2022 an informal consortium of UK animal-directed organisations that fund research on canine health and welfare commissioned an external analysis of this sector. This project was originally conceptualised as an audit of research funding by these and other similar organisations to identify topic gaps in previous funding distribution and to facilitate the development of a coherent and evidence-based collaborative strategy for improved future funding decisions. However, wide-scope funding bodies, such as the UKRI councils, also provide significant grants for canine-relevant research, often contributing to research initiatives that are also funded by animal-directed organisations [8,9]. Consequently, the current study was expanded to additionally harvest and analyse available data from these wide-scope organisations, to situate the funding from animal-directed organisations within the broader context of UK not-for-profit canine health and welfare research funding [10]. Therefore, this study aimed to quantitatively map and qualitatively analyse the overall landscape of UK-funded not-for-profit canine health and welfare research between 2012–2022.

## 2. Methods

### 2.1 Data selection methodology: Definition of in-scope funding organisations

This research project was commissioned by an informal consortium of animal-directed UK-based funders. Before the project began, seven animal-directed organisations (Battersea, BSAVA PetSavers, BVA Animal Welfare Foundation (BVA AWF), Dogs Trust, Kennel Club Charitable Trust (KCCT), PetPlan Charitable Trust and Waltham Foundation) had already committed to contributing their historical research funding data, spanning 2012–2022.

Additional in-scope funders were defined as “*any UK-based funder that issues external research grants on a not-for-profit basis which are primarily or subsidiarily intended to advance knowledge relevant to canine health and/or welfar*e”.

Inclusion and exclusion criteria for funding sources:

- Geographical location: Grants awarded by overseas funders for work carried out in the UK were excluded from the current analysis, but grants awarded by UK funders for work carried out partly or entirely overseas were included.
- External vs. internal funding: This study only included projects where external funding was awarded, and thus ‘in-house’ research was excluded, despite its considerable significance within some organisations (e.g., several in-scope funders of external research, such as Dogs Trust, Guide Dogs and The Kennel Club, also employ salaried researchers and produce scientific papers using data generated from ongoing internal activities). Similarly, research at veterinary schools arising from in-house clinical work was excluded. Research grants issued by veterinary corporate groups were also excluded, because these grants were often exclusively or preferentially available to internal applicants, so that it was difficult to apply clearcut standardised inclusion criteria across all providers in this group [11–14]. Research undertaken by corporate or referral veterinary practices was included when it involved the receipt of a grant awarded by an external in-scope not-for-profit funder.
- Profit: As this study focused upon not-for-profit research, commercial research funded by organisations such as pharmaceutical companies was excluded from this analysis, except when such research involved the receipt of a grant awarded by an in-scope not-for-profit funder.
- Financial vs. in-kind contributions: Only direct financial research support from owner communities was included in these data, with other types of in-kind support, e.g., owner communities sourcing dogs for participation in breed-specific or problem-specific research projects, excluded from the dataset.

### 2.2 Data collection process

Data were collected by arrangement from the animal-directed organisations that had commissioned the project, and a representative from each organisation described its funding decision process. Additional comparable animal-directed UK charities, including the Blue Cross, PDSA and RSPCA, were also invited to participate. Other animal-directed organisations, such as veterinary school charitable trusts, veterinary specialist societies and other relevant small societies, were similarly contacted individually where possible.

Data from wide-scope funders (Wellcome Trust, UKRI councils) were harvested through keyword searches of publicly available grant data on organisational websites [15,16] This harvested dataset has been made available at https://doi.org/10.6084/m9.figshare.25353301.v1. A similar approach was used to survey the Charity Commission website to identify other canine-relevant funding bodies, which were then individually approached if in-scope [17]. Historical information about the Animal Health Trust, a major centre for UK research into canine health until its closure in 2020, was obtained through its archive at RCVS Knowledge and through direct contact with some of its former staff [18].

This outreach was supplemented by publicity in the veterinary press [19], through social media and through personal networks, to raise awareness of the project and the request for funding data within the veterinary and canine research sectors. In parallel, the project was also advertised to dog breeders via the show dog press [20], The Kennel Club’s breed health coordinator network (a list of volunteers with a particular interest in breed health, who liaise with each breed community) and personal contacts, to request data sharing on breed community funding of research into breed-specific health problems.

### 2.3 Methodology for selection of in-scope research projects

Data obtained from in-scope funding organisations were scrutinised to identify in-scope research projects. In-scope (canine-relevant) research projects were defined as *“those supported from 2012–2022 by external not-for-profit grants provided by UK-based funders*, *and which were primarily or subsidiarily intended to advance understandings of canine health and/or welfare*”. Grants awarded by animal-directed funders were excluded if the project description did not include dogs at all (for example, grants concerned only with cats or horses). An alternative selection strategy was required for wide-scope funders such as UKRI councils, because their in-scope projects had to be extracted from a large public database which also included many out-of-scope projects. For each of these organisations, a shortlist of potentially in-scope projects was generated by searching its public database using suitable keywords, such as ‘dog’; ‘dogs’; ‘canine’; ‘canid’; ‘breed’; ‘pet’; ‘pets’; ‘companion animal’. After excluding obviously spurious inclusions, such as linguistic studies which used ‘dog’ as a vocabulary word in their project descriptions, the fuller records of remaining studies were scrutinised to determine their relevance to domesticated canine health and welfare. Relevance was evaluated using the following definition, which developed through repeated iterations of this process:

“*In-scope research projects explicitly refer to domesticated dogs*, *either specifically or through a canine-inclusive term such as ‘companion animal*’. *Most in-scope projects involve the active study of actual dogs*, *their body tissues*, *or human perceptions of them*. *In-scope projects explicitly or contextually have the potential to advance understanding of canine health*, *disease*, *demography*, *physiology*, *genetics*, *behaviour or welfare*, *or of the human-animal bond*, *even if their primary intent is to improve human health or advance knowledge in other ways*”.

This overarching definition applied to all research grants that were deemed canine-relevant. It comprised four inclusion categories with differing criteria.

1. Grants which supported the **direct study** of actual dogs, their body tissues, or human perceptions of them–for example, veterinary clinical studies, laboratory studies using canine cell lines, or surveys of human attitudes to dogs.
2. Grants which did not involve the direct study of dogs, but which were explicitly intended and framed to offer **benefit to dogs** as a major outcome–for example, studies to develop alternative laboratory disease models that reduced or replaced the laboratory use of dogs.
3. Grants which supported research that investigated pathogens which are major specific causes of canine disease, such as leptospirosis or rabies. These were considered **contextually canine-relevant** even if the available information did not indicate that the research directly involved dogs or their biological tissues.
4. Grants where the investigators had explicitly positioned their research as offering potential benefit to companion animals. In these cases, **researcher framing intention** was used as an inclusion criterion, even if the available information indicated that the research did not directly involve canine biological material.

These inclusion criteria were iteratively developed by the project lead (AS) during the main period of data collection and ratified through discussion with DO’N and RMAP. Most inclusion decisions were made by AS, using the scope definition and inclusion criteria as described. In a few equivocal cases, the decision was finalised through group discussion.

### 2.4 Supplementary data harvesting

Harvested sets of in-scope data varied greatly in their level of detail between (and in some cases within) funding organisations, ranging from full multi-page project descriptions to simple lists comprising just the project title, year and amount funded. All in-scope grants were tabulated in Microsoft Excel for Office 365 using this signalment information, including descriptors such as the funding organisation and research institution/s. Where fuller project information was lacking, this was supplemented through exhaustive internet searches, which used sources such as university websites, Google Scholar, LinkedIn and key phrase searches to identify researchers, find project outputs such as published papers, and gain an overview of unfamiliar research methodologies, to synthesise a fuller understanding of each research project.

### 2.5 Data analysis

#### 2.5.1 Qualitative content analysis: Primary coding

A qualitative content analysis (QCA) approach was used to conduct an analysis of in-scope projects. QCA captures key meanings from unstructured or variable source material (such as interviews, speech or original documents) in a structured, rigorous way, through an iterative multi-step analytical process, which aims to draw out key aspects of the source material while reducing its volume [21–24]. In the current project, QCA was used to extract and categorise content that was explicit or immediately implicit in the material (for example, research into hip replacement techniques could be coded as relevant to orthopaedics and breed-related disease). Therefore, a combination of inductive category formation and deductive category assignment was adequate to summarise the material effectively [22].

For efficient reference, each project topic was summarised to one term–for example, ‘Developing phage cocktails tailored to treat particular urinary infections’, or ‘Fine mapping of genetic markers of obesity in pugs’. This summarised term, along with the project abstract or detailed research methodology, where available, was used to generate a list of relevant keywords for each project, which combined to comprehensively capture key aspects of the research, such as its subject, approach, and methodology. The datasets from three funding bodies (KCCT, Waltham Foundation and Wellcome Trust) were mapped in this way. The generated keywords were then extracted, amalgamated to remove duplicates and homologues, and organised into different categories, such as ‘breed of dog’, ‘clinical speciality’, ‘research setting’. Following QCA techniques, these categories were thus created iteratively as the keywords were examined and consolidated, rather than having been predetermined beforehand. Category formation continued until all keywords had been assigned a category or amalgamated with others.

This process created twelve categories or dimensions of content analysis (Table 1), which were then used to create a customised coding frame in Microsoft Excel, compiled with the basic project reference data described previously. When creating the coding frame, these dimensions of analysis were provided with a menu of category choices derived inductively from the keyword lists and extended deductively from prior knowledge if required (for example, including further clinical and non-clinical fields in the ‘specific knowledge field’ dimension). One-to-many coding was employed for some dimensions of analysis, so that up to six relevant categories could be selected where appropriate. For example, research projects that investigated candidate genes for inherited eye diseases were coded for both ‘ophthalmology’ and ‘molecular genetics’ in the ‘specific knowledge field’ dimension. Following standard QCA practice, approximately 10% of the total cleaned data were then entered in the coding frame by one researcher (AS) and cross-checked by the other researchers (DO’N and RMAP). Once the coding frame and process had been validated in this way, it was used to re-code the pilot datasets and extended to other datasets as they were collected.

**Table 1:** List of categories/dimensions used in canine research funding dataset content analysis, with example keywords.

Categories/dimensions of content analysis	Example keyword choices
Broad focus	Canine focused; One Health
Canine niche	Free-living; owned
Knowledge sector	Humanities/social science; veterinary/medical
Specific knowledge field	Canine behaviour; dermatology
Output	No results found; peer reviewed paper
Breed category	All dogs; brachycephalic breeds
Canine role	Biological material; living animal
Research setting	International fieldwork; laboratory
Research approach	Interventional/comparative audit; observational/descriptive analysis
Type of problem	Breed-related; infectious
Human role	Pet owner, shelter worker
Interventions studied	Gene mapping, surgical procedure

Two QCA dimensions developed in this coding process require further description. One coded the database according to the **broad focus** of each research award, dividing projects into five non-overlapping categories. These were defined as:

1. **Canine-focused**—where dogs were the primary subject of investigation
2. **Animal-focused**—research that considered dogs alongside other non-human species
3. **Human medicine**—where dogs were deployed as models for human diseases
4. **One Health** research
5. **Other** approaches, mostly investigations in humanities or social science.

The other QCA dimension developed in this coding process which requires further explanation was a metric to assess grant outcomes. This was devised because the ultimate research output is an important consideration in prospective funding decisions. It was not possible to fully assess the final impact, value or implementation of research outputs, because these parameters would be too variable, subjective and nebulous to feasibly capture in a meaningful or standardised way across the whole dataset. Therefore, while recognising that not all projects are equally likely to produce publications and that there are inherent difficulties in correlating publication data with research impact, it was nevertheless decided that a simple measure of publication output was the most useful and achievable metric of analysis for the current study [25]. Using a range of investigative approaches, each research project was coded to indicate what type of published results it had produced, if any, using categories such as ‘at least one peer reviewed paper’ (it was impractical to count how many), ‘public results’ (which included conference proceedings and written reports), ‘thesis’, or ‘no output traced’. Each grant was coded according to the most rigorous publication method that was identified for it. Projects with current or very recent (<3 years) funding, or where other information indicated that research was still ongoing, were coded as ‘recent’, unless a public output was identified. Non-recent projects whose funding had terminated no more than five years ago, or where the termination was unclear, were coded ‘uncertain’, unless an output was identified.

In addition to the predominantly inductive coding process previously described, deductive coding was used to also group research grants by main recipient institution, and to further group these institutions into four categories, to allow investigation of funding distribution between institutions. These four categories were:

1. UK universities with an (established) veterinary school
2. UK universities without a veterinary school
3. Other UK sites (e.g. referral practice)
4. International institutions (all types)

Grants were considered as awarded to the ‘veterinary school’ group even if they were awarded to non-veterinary departments at a university with a veterinary school, because research was often multidisciplinary and explicitly conducted by several departments working together. Even when that was not the case, the presence of a veterinary school within a university arguably influenced the historical development of expertise within that institution, as discussed later. Each organisation that was a federal member of the University of London was categorised as a separate institution, however, reflecting their separate infrastructures (for example, the RVC was coded as a stand-alone veterinary school, whereas the London School of Hygiene and Tropical Medicine was coded as a non-veterinary school UK university). Research at the Animal Health Trust was coded as ‘UK veterinary school’, both because as a specialist centre of veterinary research it effectively functioned as a non-teaching veterinary school, and because after its closure its canine research activities were largely transferred to the University of Cambridge.

#### 2.5.2 Qualitative data analysis: Secondary coding of key research topics

This overall coding process created a flexible tool, which could have been used to answer a multitude of specific questions of niche interest, such as ‘who has funded research into breed-specific disease in Labradors?’; or ‘how much money was spent on orthopaedic research in 2016?’ However, it was decided that a wider lens of analysis would provide results of broader interest across the canine health and welfare sector. The QCA coding frame previously described was therefore subsequently used to compare the funding of several key research topics between funding sectors and institutions, thus providing a baseline for use in future comparative work.

Eight topics were chosen for this element of the current analysis, selected for their broad relevance across the canine health and welfare sector. These were:

1. **Antimicrobial resistance (AMR)**. A topical issue of great concern across the veterinary sector, including companion animal medicine [26,27].
2. **Breed-related disease (BRD)**. A topic of particular interest to the canine health sector. This was taken to include all diseases where breed is a strong predisposing factor, including both inherited disease and conditions related to body shape [28,29].
3. **Conformation-related disease (CRD)**. A subset of BRD, this was taken to mean ongoing chronic disease directly related to canine structural morphology: brachycephaly and its associated defects; Chiari-like malformation/syringomyelia (CM/SM); intervertebral disc disease; and joint dysplasia. Other types of breed-related disease which are more sporadic in their development and less directly linked to body shape, such as osteosarcoma or dilated cardiomyopathy, were excluded.
4. **Canine genetics**. A topic with obvious overlap with breed-related disease. However, not all BRD research involves genetics; nor does all canine genetics research involve BRD, as much of it concerns diseases that are not strongly linked to certain breeds. All research that advanced understandings of any aspect of canine genetics was included, but projects which studied the genome of pathogens rather than the genome of the dog were excluded.
5. **Neoplasia**. A common problem of concern in canine medicine [30].
6. **Clinical relevance**. A subject of particular interest to clinically active researchers, who may want to know which funders support this type of research. For this analysis, clinically relevant grants were defined as those directly related to advancing the diagnosis, prevention or treatment of physical or mental disease in non-experimental dogs. Theoretical modelling of disease transmission, social science research or research involving secondary data, and the preliminary (pre-clinical) development of new therapeutic or diagnostic interventions were generally excluded, unless they were framed as having direct relevance to clinical work. For example, biological tissue studies to investigate pathological processes were excluded whereas research to pilot the diagnostic use of new biomarkers was included; most electronic data surveillance research was excluded, but e-clinical trials that were intended to directly inform treatment protocols in practice were included.
7. **Human-animal interactions** (HAI). This was coded as a broad category to include all approaches to the human-animal relationship, ranging from theoretical work in the humanities, through social science, to practical studies of human/canine behaviour and interactions.
8. **Canine behaviour and welfare**. Much HAI research prioritises the human side of the relationship, either to investigate how canine-related hazards to people can be mitigated (as with rabies or dog bites) or to explore how people may benefit from their relationships with dogs. However, funders may want to prioritise work that foregrounds the dog rather than the person. Therefore, the overall dataset was also explored to investigate funding patterns that supported practical research into canine behaviour and welfare. This was taken to mean studies where actual dogs, or people’s perceptions of them, were the subject of investigation, with the direct intent of advancing knowledge about some aspect of canine cognition, behaviour or welfare.

### 2.6 Research impact metrics: ‘Benefit for the dog’ and ‘pathway to impact’

Each project was also evaluated on two further criteria: ‘benefit for the dog’ and ‘pathway to impact’. ‘Benefit for the dog’ was intended to capture the potential level of benefit of any given project for dogs themselves, rather than for other potential beneficiaries, such as other species, abstract knowledge, or the environment. ‘Pathway to impact’ was intended to capture how directly and readily each project could be expected to produce this ‘benefit for the dog.’ Numerical scoring systems were devised for both metrics to grade each project included in the study to facilitate comparisons within the dataset.

#### 2.6.1 Benefit for the dog

Previous research which scored and compared the canine welfare burden caused by different types of problem identified prevalence, duration and severity as key elements for consideration [28,31,32]. Following this principle, ‘benefit for the dog’ was evaluated through two scales: (1) number of dogs benefiting and (2) impact on individual welfare. To indicate the approximate maximum number of dogs globally that could be expected to benefit from the research if its aims were achieved, a five-point scale was devised (Table 2). Estimating the number of dogs potentially affected by the health or welfare issue addressed by each research project involved collating various sources of information, such as online searches, breed registration data and academic publications, to identify relevant factors, such as breed population size for breed-specific inherited diseases, or the overall prevalence for infectious or acquired diseases such as osteoarthritis. This score was then modified according to whether the research was intended to advance knowledge in a way that could benefit many dogs with that condition, or to develop an intervention that would only ever be available to a minority of animals. For example, a new drug for the treatment of osteoarthritis might potentially benefit millions of dogs with joint pain, whereas a new joint replacement technology could be expected to only benefit far fewer individuals.

**Table 2:** Benefit for dog: Scoring table for numerical metric.

Number of dogs that may benefit (international)	Examples of problem	Numerical score
Millions	Arthritis; obesity; parasite control	5
(tens/hundreds of) Thousands	BOAS; MVD; shelter husbandry; drug trial	4
Hundreds or fewer	Rare disease gene test; pioneering surgical treatment	3
Indirect benefit only	Human HAI benefit; laboratory dog as human disease model	2
No apparent benefit	Philosophy of HAI; canine archaeology	1

Secondly, projects were assessed to consider the significance of the problem addressed by the research in terms of its impact on canine welfare for each affected dog. This was scored on a six-point scale (Table 3), which aimed to encompass both severity and duration of the problem addressed. Some health conditions (e.g., syndromic conditions such as BOAS) can show wide clinical variability *within* the affected population of dogs. For these disorders, the expected impact of each problem on a ‘typical’ affected dog was estimated, to better represent the overall impact on the population, which will include dogs with more mild disease as well as more severe disease. This also prevented the overall scale across all projects from being unduly skewed towards the most serious outcomes.

**Table 3:** Benefit for dog: Scoring table for impact on wellbeing metric.

Impact of condition or problem on dog’s wellbeing (includes severity and duration for affected dog)	Examples of problem	Numerical score
Severe impact, typically rapidly fatal	Rabies; myelomalacia	6
Severely life-restricting, often fatal	Osteosarcoma; DCM; severe aggression	5
Typically serious and/or lasting impact	BOAS; MVD; severe separation anxiety	4
Typically moderate impact	Atopy; obesity; arthritis	3
Typically minor or temporary impact	Surgical neutering; acute diarrhoea	2
Little or no impact	Normal cognition; normal biomechanics	1

Number of dogs benefiting and impact on wellbeing scores were multiplied together to produce an overall ‘benefit for the dog’ score ranging from 1 to 30. A multiplied score was chosen in preference to an additive score, to create a scale giving greater weighting to common problems that also have a major welfare impact [33]. An additive scale could have placed undue emphasis on serious conditions that only affect very few dogs, such as an inherited disease specific to a rare breed.

#### 2.6.2 Pathway to impact

A ‘pathway to impact’ metric was devised to represent the potential for implementation of the intended outcomes from a research project. This can be considered in terms of the potential **effectiveness of the proposed intervention in an ideal world** and the actual **logistics of its implementation in the real world**, which includes the time, cost, difficulty, political acceptability and feasibility of its enactment. These were separated into two scales, because some theoretically effective interventions can be difficult to achieve (for example, discouraging people from breeding or purchasing dogs with extreme conformation).

The theoretical effectiveness of a research output will inherently vary because some research topics are more solution-focused than others. For example, the development of a new drug has a direct, physical, intended end-product, whereas research that explores attitudes to canine euthanasia does not. Therefore, a five-point ‘ideal world impact’ scale was developed to measure this metric (Table 4).

**Table 4:** Pathway to impact: Scoring table for ideal-world impact metric.

Ideal-world impact of research findings	Examples	Numerical score
Immediate direct canine benefit	Gene test; clinical trial; improved environment in rescue centres	5
Direct canine relevance but no immediate implementation	Finding a disease biomarker; sociology of a welfare problem; sequencing neoplastic genomes	4
Implementation to benefit other species, does not directly benefit dogs	Environmental impact of canine parasiticides; human benefit from canine therapy	3
Research advances knowledge but no direct practical intervention	Sequencing archaeological canine genomes; studying a normal biochemical process	2
Limited scope or flawed design, significant output unlikely	Research trivial or poorly designed	1

Attempting to quantify the real-world logistics of implementing a research output was subject to additional challenges. Although funders can request a detailed forecast of real-world logistical factors that enhance or create barriers to impact as part of a grant proposal, it was seldom possible to recover or recreate this information from historical project funding data, and often impossible for an observer to surmise what these factors might be, particularly for highly technical fields. Therefore, a proxy metric of ‘feasibility and scope of human behavioural change’ was developed for the purpose of the current analysis, based on expected human behaviour change resulting from the project (Table 5). These two scores were added together to create an overall scale from 2 to 11 to represent ‘pathway to impact’. An additive score was considered more appropriate for this metric.

**Table 5:** Pathway to impact: Scoring table for real-world feasibility metric (represented by human behaviour change (HBC)).

Feasibility and scope of human behavioural change (HBC)	Examples	Numerical score
Direct route to HBC, within control of funder or linked organisation	Dogs Trust changes its rehoming or kennelling policies; KC changes its engagement with breed-related disease	6
Clear route to HBC, with few barriers to adoption within relevant sector	New gene test; clinical trial for new drug	5
Structural barriers to HBC–findings widely accepted but implementation tricky	Epidemiology of endemic zoonotic disease; AMR	4
Political barriers to HBC–findings potentially controversial	Conformation-related disease in pedigree dogs; health issues with international rescue dogs	3
Technical/professional HBC only–no public HBC expected	Most laboratory research; investigation of veterinary workplace practices	2
No behavioural change expected	Very theoretical or poorly designed research	1

### 2.7 Quantitative data analysis

Quantitative data analysis was carried out using Microsoft Excel for Office 365 (version 2311) and IBM SPSS version 29 software. Data were coded in Excel and exported to SPSS for statistical analysis as appropriate. The multiple individual sums contributed by separate breed communities to the Animal Health Trust’s 2016 ‘Give a Dog a Genome’ (GADAG) project were generally collapsed into one data point, as this was one overarching project with effective ‘crowdfunding’ [34]. This created a modified dataset with 608 grants for quantitative analysis. Continuous (numerical or financial grant-related and scoring metric) data were assessed for normality by visual scrutiny of histograms. Non-normal results were reported as medians (interquartile range (IQR) and range). Univariable analysis used the chi-squared test to assess for association between categorical variables (for example, grant output type and funding sector). The Mann-Whitney U test was used to assess for association between categorical variables and non-normal continuous variables (for example, funding sector and ‘benefit for dog’ score). A p value of <0.05 was considered significant [35].

## 3. Results

### 3.1 Overall summary

In total, information was collected on 684 research grants awarded between 2012 and 2022 from 109 funding sources (Table 6). Some projects received multiple grants from one or more funders, and some projects were linked to other projects concurrently or successively, so that the precise number of separate research projects depended on how the term ‘project’ was defined. However, over 500 separate research projects were identified. Funding bodies fell into three categories: wide-scope funders, comprising multiple UKRI councils and the Wellcome Trust (10 organisations: this data is available at https://doi.org/10.6084/m9.figshare.25353301.v1; various general animal-directed charities and other organisations, all concerned with veterinary and/or animal welfare activities (18 organisations); and various breed-specific community fundraising groups and charities (81 breeds).

**Table 6:** Not-for-profit UK canine health and welfare research funding summary, 2012–2022.

Organisation	No. in-scope grants awarded	Percentage of in-scope grants awarded	Total in-scope funding	Percentage of total in-scope funding
**Wide-scope funders**				
BBSRC	66	9.6%	£18,907,671.00	32.7%
Wellcome Trust	17	2.5%	£7,239,461.00	12.5%
MRC	10	1.5%	£4,829,173.50	8.4%
Other UKRI councils (< £3 million each)*	52	7.6%	£10,188,900.00	17.6%
**Subtotal**	145	21.2%	£41,165,205.50	71.2%
**Animal-directed funders**				
Dogs Trust	81	11.8%	£6,955,661.17	12.0%
KCCT	51	7.5%	£3,952,356.03	6.8%
PetPlan Charitable Trust	87	12.7%	£2,805,446.90	4.9%
BSAVA PetSavers	97	14.2%	£941,218.09	1.6%
Waltham Foundation	28	4.1%	£537,063.00	0.9%
Other funders (< £500K each)**	72	10.5%	£1,061,580.48	1.8%
**Subtotal**	416	60.8%	£16,253,325.67	28.1%
**Breed communities**	123	18.0%	£370,431.53	0.6%
**TOTAL**	**684**	100.0%	**£57,788,962.70**	100.0%

* Other in-scope large-scale funding councils, < £3 million funding identified: AHRC, EPSRC, ESRC, Innovate UK, NC3Rs, NERC, UKRI

** Other in-scope animal charities, < £500K funding identified: AHT, Battersea, Blue Cross, BVA AWF, CamVet, Guide Dogs, Langford Veterinary Services Clinical Research Fund, RSPCA, RVC ACT, SCAS, SSPCA, UFAW, Wood Green.

In total, these 28 larger organisations and 81 breed groups provided traceable funding of £57.8 million during the period that was surveyed. Wide-scope funders made canine-relevant awards that totalled £41.2 million (71.2% of total funding); animal-directed organisations made canine-relevant awards that totalled £16.3 million (28.1% of total funding); and breed-specific groups provided funding of £370K (0.6% of total funding). Individual awarded grants ranged in size from £300 to £2.3 million.

These data omit some in-scope funding, as it was not possible to contact all in-scope funders. Some had no clear point of contact; others did not respond to repeated requests for information. This was particularly true for breed communities. Only 30 breed community representatives directly responded to this project’s call for data, although 222 breeds are currently registered with The Kennel Club and the majority of these breed communities are represented by health coordinator volunteers who were sent project information both via The Kennel Club’s health team and via social media (a private Facebook group) [36]. Although some further breed-specific data were harvested through other means, this discrepancy suggests that the breed community research funding captured in this project is a significant underestimate. Moreover, some breed communities and other smaller funders, such as small charities, retained few or no records of past funding decisions. Some larger funders also provided incomplete data, particularly for earlier years of the period under analysis.

### 3.2 Funding patterns over time

**Fig 1:**
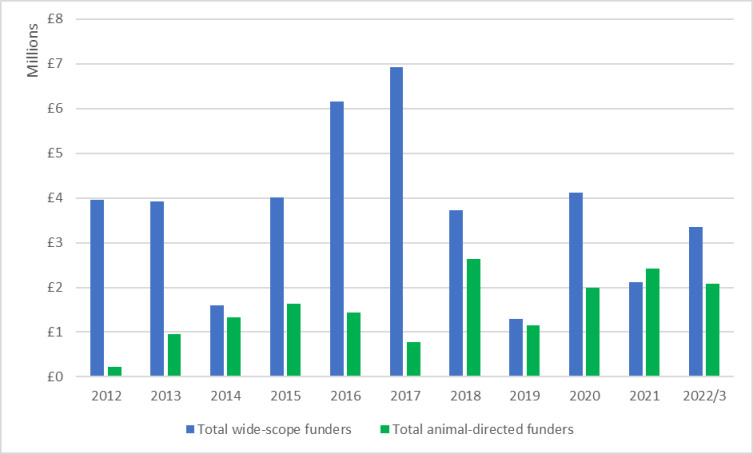
Annual UK canine-relevant funding categorised by wide-scope and animal-directed funders, 2012–2022.

The £57.8 million of total funding identified from 2012–2022 was distributed unevenly over time, as shown in Fig 1. All data reflect the actual funding awarded without considering inflation. Breed community funding is here subsumed into other animal-directed funding. Although the graph demonstrates that wide-scope funders overall contributed more than twice as much money as animal-directed funders, no apparent temporal trend is clear within these data. For example, total annual funding peaked at £7.7 million in 2017; minimum total annual funding was £2.5 million, in 2019. This fluctuation was largely caused by the variable awarding of single large grants by wide-scope medical funders. The 2017 peak included a Wellcome Trust grant of £2.3 million for a One Health project on rabies and an MRC (Medical Research Council) grant of £1.5 million for a One Health study of diabetes mellitus, so that just two awards accounted for almost half the total annual funding captured for that year.

Other incidental variation contributed to fluctuation in annual funding. Many wide-scope funders only appeared intermittently in the data. For example, NERC (the National Environment Research Council) awarded £2.2 million for three canine-relevant projects in 2016 but provided no canine-relevant funding in 2015 or 2017. Canine-relevant funding from animal-directed organisations fluctuated because of variable support for out-of-scope projects, such as those concerning the cat.

### 3.3 Wide-scope versus animal-directed funders

Less than a third of overall canine-relevant funding originated from the animal-directed sector, whereas over 70% was provided by wide-scope funding bodies. However, as shown in Table 6, animal-directed organisations, excluding breed communities, numerically awarded roughly three times as many canine-relevant grants as wide-scope funding bodies, because wide-scope funding bodies typically provided much larger grants. The median grant from wide-scope funders was £267K (IQR £98K - £442K, range £3K - £2.3 million), representing 120 funded projects (excluding 25 PhD awards where funding details were not disclosed). In contrast, the median grant awarded by animal-directed organisations was £10K (IQR £5K - £38K, range £300 - £546K) but represented 416 awards. Awards from individual animal-directed organisations were thus typically less than a tenth the value of those made by individual wide-scope funders. Breed community funding awards were typically even smaller, with a median grant size of £1K (IQR £1K - £2.5K, range £500 - £28K), calculated from 123 awards. ‘Crowdfunding’ contributions to the Animal Health Trust’s 2016 ‘Give A Dog A Genome’ project accounted for 78 of these awards, which were collapsed into one data point for most analyses in this study.

Animal-directed research funding differed from wide-scope funding in ways other than the financial grant size. None of the animal-directed organisations that provided data for this study normally funded canine research that required a Home Office licence [37], with some imposing further restrictions, such as not funding research that involves surgical procedures, and all explicitly or implicitly required that funded research should directly concern animal health and welfare or the human-animal bond. In contrast, wide-scope funders funded many projects which, although canine-relevant, generally did not seek to foreground the dog but instead aimed to support the investigation of problems that affect canine health or welfare from a broader One Health or human medical perspective. Moreover, several wide-scope funders, such as BBSRC, did fund projects that required a Home Office licence.

#### 3.3.1 Broad focus coding

**Fig 2:**
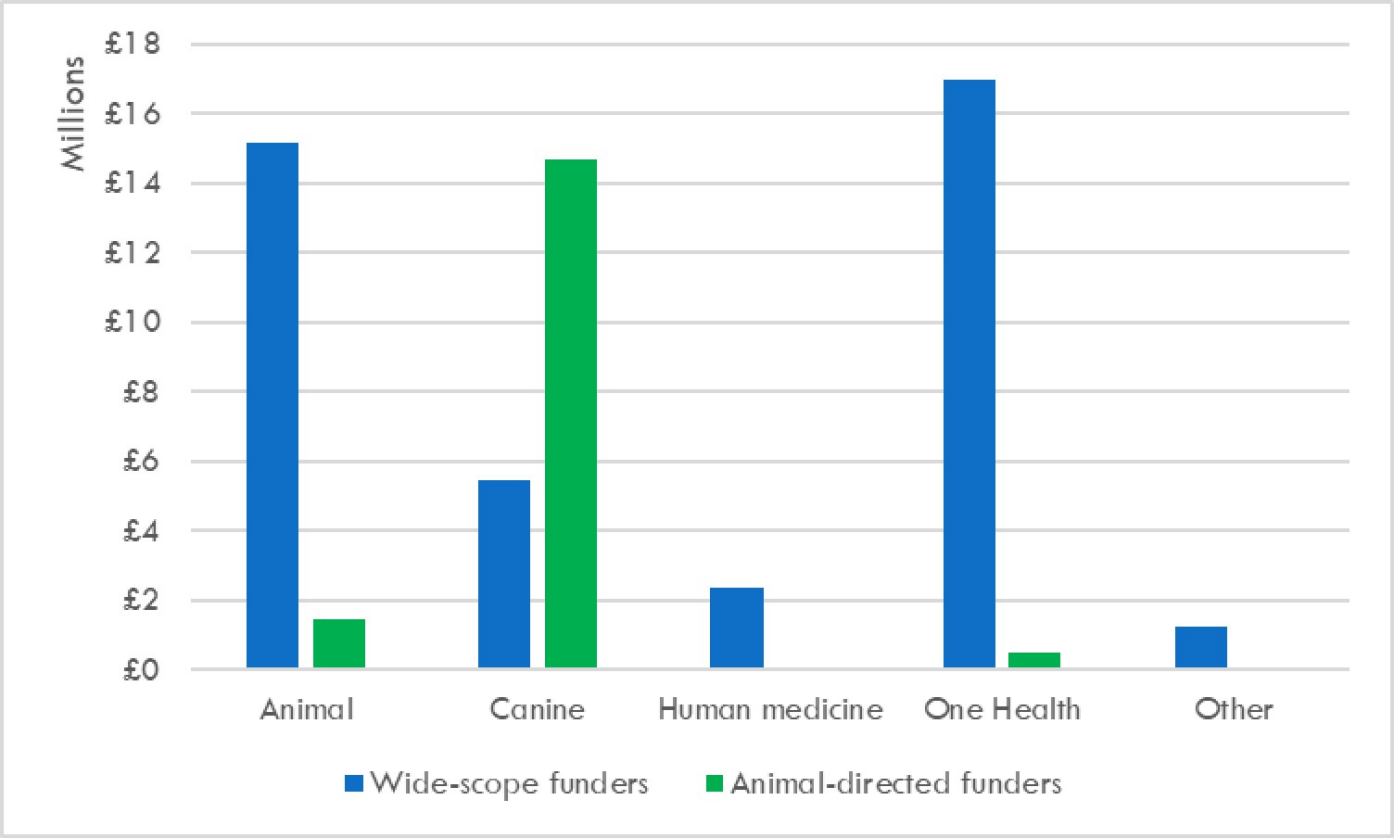
UK canine-relevant funding categorised by type of funder and by broad category, 2012–2022.

To explore this distinction further, as previously described (see Table 1), one QCA dimension in this study coded the database according to the broad focus of each research award, dividing projects into five categories: canine-focused; animal-focused; human medicine; One Health; and other. Using these categories, projects that were funded by wide-scope organisations were compared to projects funded by animal-directed organisations, with breed community funding here subsumed into other animal-directed funding (Fig 2). Wide-scope funders showed very different patterns of support for research related to canine health and welfare when compared to animal-directed funders. Canine-relevant funding from wide-scope funders covered (albeit unevenly) research in all five of these categories, and so had a much broader scope than that from animal-directed organisations, which largely funded work framed directly around dogs, with some smaller funding for work also concerned with other animals or (rarely) One Health. For example, about 6% (£2.2 million) of canine-relevant funding from wide-scope funders was primarily directed towards human medicine. The largest grant in this category was a 2013 Wellcome Trust award for just under £1 million, aimed at developing a canine laboratory model for Duchenne muscular dystrophy in humans. Work of this type requires a Home Office licence [37] and is ineligible for support from animal-directed funders. Wide-scope funders also supported five canine-relevant projects, totalling £1.2 million, categorised as ‘other’. These projects investigated non-biological subjects such as interspecies geographies and concepts of rationality; animal-directed funders instead prioritised more pragmatic investigations. However, the majority (78%) of canine-relevant funding from wide-scope funders supported the two categories of One Health and animal-focused research. In contrast, 73% of funding from animal-directed funders supported canine-focused research.

#### 3.3.2 One health research

Canine-relevant projects that were explicitly or implicitly presented as One Health research attracted £17.5 million of funding in total between 2012 and 2022, comprising 30% of all canine-relevant research funding (Fig 2). The majority (97%) of One Health funding was provided by wide-scope organisations, accounting for 41% of all wide-scope funding. Most One Health research, across both wide-scope and animal-directed funders, could be grouped into two categories: zoonotic disease and ‘one medicine’. The investigation of various zoonotic infectious diseases accounted for £9.2 million of wide-scope One Health funding, with rabies (£3.5 million) and multi-species investigations of antimicrobial resistance (£2.5 million) as major recipients. ‘One medicine’ received £7.3 million of funding. This term is used here to denote investigations of biological processes or technologies that were framed as offering mutual benefit for dogs, humans and sometimes non-canine animal species. For example, the three biggest ‘one medicine’ awards were a 2017 MRC grant of £1.5 million for a multispecies study of the genetics of diabetes mellitus in dogs and people, and twin 2018 grants, totalling £1.5 million, awarded by the MRC and BBSRC for a One Health investigation into panvalent morbillivirus vaccine technology. A much smaller proportion of wide-scope One Health funding, totalling £447K, was directed to projects that used a multispecies medical humanities perspective to explore medical and veterinary issues within the same conceptual framework. The largest grant in this group was a 2020 Wellcome Trust award for £286K, which compared medical and veterinary approaches to end-of-life care. Animal-directed funders provided £502.5K of One Health funding: of this, £359K supported research into zoonotic disease, £138K supported ‘one medicine’ research, and £5.5K concerned a project that investigated the environmental impact of companion animal parasiticides.

#### 3.3.3 Animal-focused research

Animal-focused research, which considered dogs alongside one or more other (usually mammalian) non-human species, accounted for 29% of all identified canine-relevant research funding (£16.6 million). Most (91%) of this funding was provided by wide-scope funders (£15.1 million), while animal-directed funders provided 9% of these funds (£1.4 million) (Fig 2). Animal-focused projects supported by animal-directed funders almost always involved dogs and other companion animal species, usually the cat. These projects were broadly similar to many canine-focused research projects, concerning topics such as vaccination, veterinary workplace practices, antimicrobial resistance and the human-companion animal bond. Animal-focused funding provided by wide-scope funders was much more varied in its scope. Dogs were studied alongside other animal species ranging from mice (comparative laboratory models for preclinical drug testing) to Tasmanian devils (the genetics of transmissible cancers). However, most animal-focused funding from wide-scope funders considered dogs alongside other domesticated species, including both companion animals and livestock, in various contexts. By far the largest beneficiary in this category was the long-running Ensembl project (https://www.ensembl.org/index.html), jointly run by the University of Edinburgh’s Roslin Institute and EMBL (European Bioinformatics Institute), which has been constructing and updating a multi-species annotated reference genomic database for domesticated animals for over a decade. This research has secured large and repeated grants from BBRSC, totalling over £6 million of animal-focused funding between 2012 and 2022. The Ensembl technology serves as a multi-species hub resource that can be deployed in many different types of research–for example, the investigation of breed-related inherited disease in the canine sector. Both Dogs Trust and KCCT have funded specifically canine-focused research projects which draw on this Ensembl technology.

#### 3.3.4 Canine-focused research

**Fig 3:**
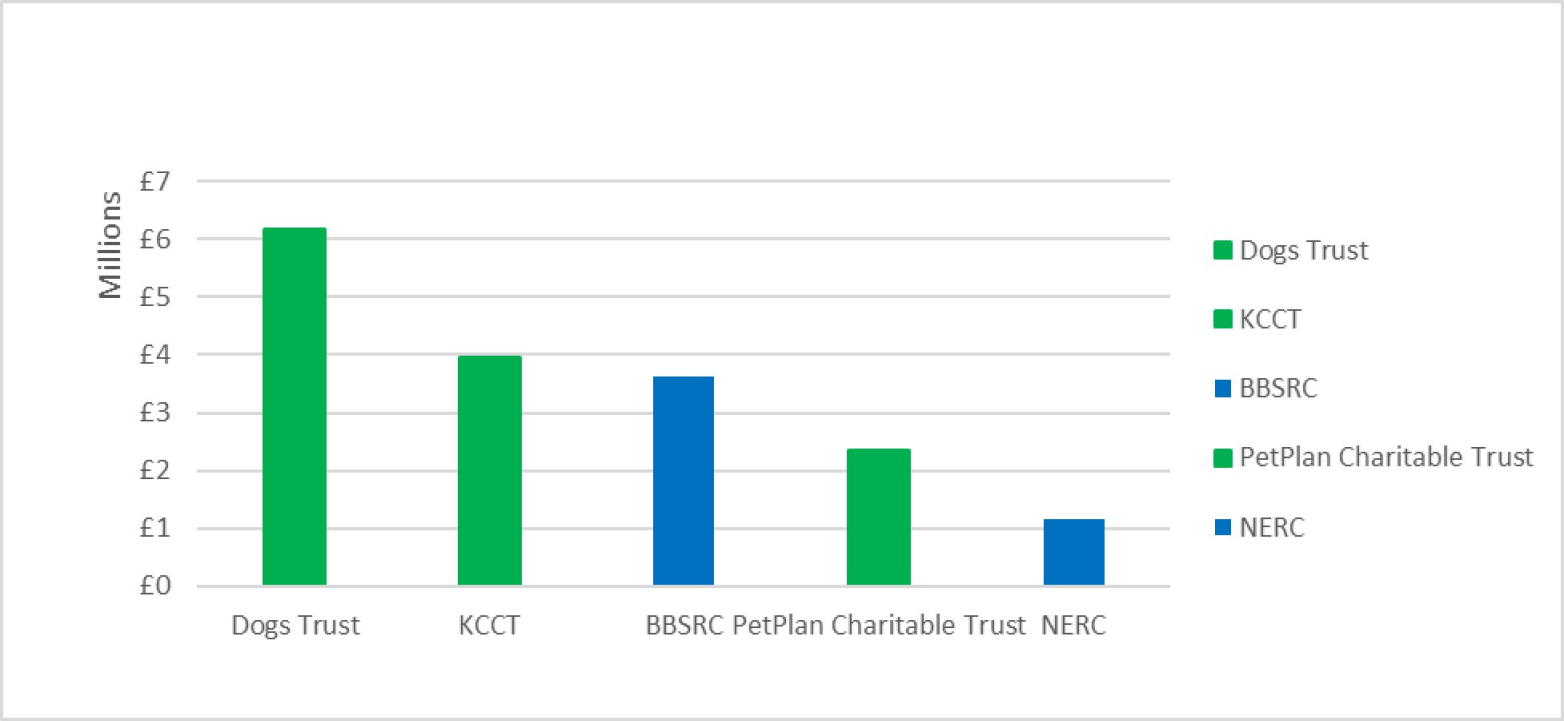
UK canine-focused funding categorised by funder, > £2million only, 2012–2022.

Combined across all funders, canine-focused research, where dogs were the primary subjects of investigation, attracted more funding than any other category, totalling £20.1 million and 35% of all identified canine-relevant funding. Wide-scope funders provided £5.4 million (27%) of this funding, while £14.7 million (73%) was provided by animal-directed funders, which thus accounted for roughly three-quarters of total canine-focused funding (Fig 2). Fig 3 depicts the five funders that provided more than £2 million of canine-focused funding. Three of these are animal-directed charities—Dogs Trust, KCCT and PetPlan Charitable Trust–which together contributed almost £12.5 million of canine-focused funding, over 60% of the total. The two major wide-scope funders of canine-focused research–BBRSC and NERC–contributed £4.8 million. However, the NERC element of this contribution, which was approximately £1.2 million, is an outlier because it consists of several grants supporting a single project that investigated the biology and geography of canine domestication. Therefore, in the mainstream landscape of canine-focused canine health and welfare research, the larger animal-directed funders and BBSRC together act as the most influential major contributors.

The canine-focused projects funded by BBSRC were biological investigations that concerned canine physiology and disease; for example, studies of epilepsy, immunology or pain perception. Other wide-scope funders (excluding NERC) together contributed £600K of canine-focused research, which was either humanities-based or concerned with developing new technologies, such as canine activity trackers. However, several wide-scope funders (such as MRC) did not fund any canine-focused research.

In contrast, the £14.7 million of canine-focused funding from animal-directed funders accounted for 88% of all canine-relevant animal-directed funding, again demonstrating the tendency of animal-directed funders to favour canine-focused projects. Some projects in this category were similar to BBSRC funded projects, investigating various canine biological and pathological processes. However, animal-directed funders also supported some research topics that wide-scope funding rarely addressed. Applied investigations of canine behaviour, social science studies of the impact of human behaviour on canine welfare, clinical audits and investigations of breed-related disease (particularly when related to extreme conformation) were overwhelmingly funded by various animal-directed organisations. Moreover, targeted research commissioned or supported by sector specialists sometimes investigated relevant questions that were otherwise entirely neglected within this dataset. For example, real-world studies that assessed the welfare impact of different management or intervention protocols on dogs in UK shelters were only funded by animal-directed organisations (see Table 7).

**Table 7:** Funding organisations and grant provision for research investigating welfare impact of management protocols for dogs in UK shelters, 2012–2022.

Organisation	Number of grants	Total amount
Battersea	1	£39,891.22
Dogs Trust	4	£150,648.73
UFAW	3	£4,360.00
Waltham Foundation	3	£53,697.00
Total	11	£248,596.95

### 3.4 Research outputs

As previously described, a QCA coding dimension (see Table 1) was developed to capture the type of research outputs (if any) resulting from each awarded grant. Visual scrutiny of a histogram that plotted number of grants with at least one peer reviewed publication against time revealed an obvious threshold between 2018 (33 identified) and 2019 (16 identified), suggesting that publication outcomes were still fluid for 2019 onwards. Therefore, data from 2019 onwards were excluded from subsequent outcome analysis. Given that the current work was conducted in mid-2023, this also highlights an issue around the temporal delay from initial funding to publication output that funders should be aware of.

The subsequent dataset contained 376 canine-relevant grants (Give A Dog A Genome was collapsed into one data point) awarded from 2012 to 2018. Of these, 194 (51.6%) led to at least one identified peer-reviewed publication, and a further 70 (18.6%) produced other public results of various types, such as a master’s or PhD thesis, a published report, or a conference presentation. Overall, therefore, just over two-thirds (70.2%) of all grants awarded during this period resulted in at least one publicly available output. There were 41 grants (10.9%) which were coded as ongoing, uncertain, or ‘other’ (for example, commercial output or ‘background’ grants to support research infrastructure, where publication was never the intended outcome). The remaining 71 grants (18.9%) had no traceable public output.

Considering the breakdown by funding sector, 96 grants (25.5%) between 2012 and 2018 were awarded by wide-scope funders, and 280 (74.5%) were awarded by animal-directed funders. Awards from wide-scope funders were significantly more likely to generate peer-reviewed publications and less likely to result in no output compared to awards from animal-directed funders (*X*^2^ = 38.87, df = 3, *p* <0.001). Of the 96 grants awarded by wide-scope funders, 74 (77.1%) led to peer-reviewed publications, and 85 (88.5%) led to at least one publicly available output. Only two (2.1%) canine-relevant grants from wide-scope funders, both of which were PhD studentships with no attributable cost, produced no traceable output of any kind. Of the 280 grants awarded by animal-directed funders, 120 (42.9%) led to peer-reviewed publications, with a total of 178 (63.6%) of projects producing at least one publicly available output. However, 69 grants from animal-directed funders (24.6%), representing a total £1.1 million of funding, produced no traceable public output.

### 3.5 Institutional distribution of research funding

**Fig 4:**
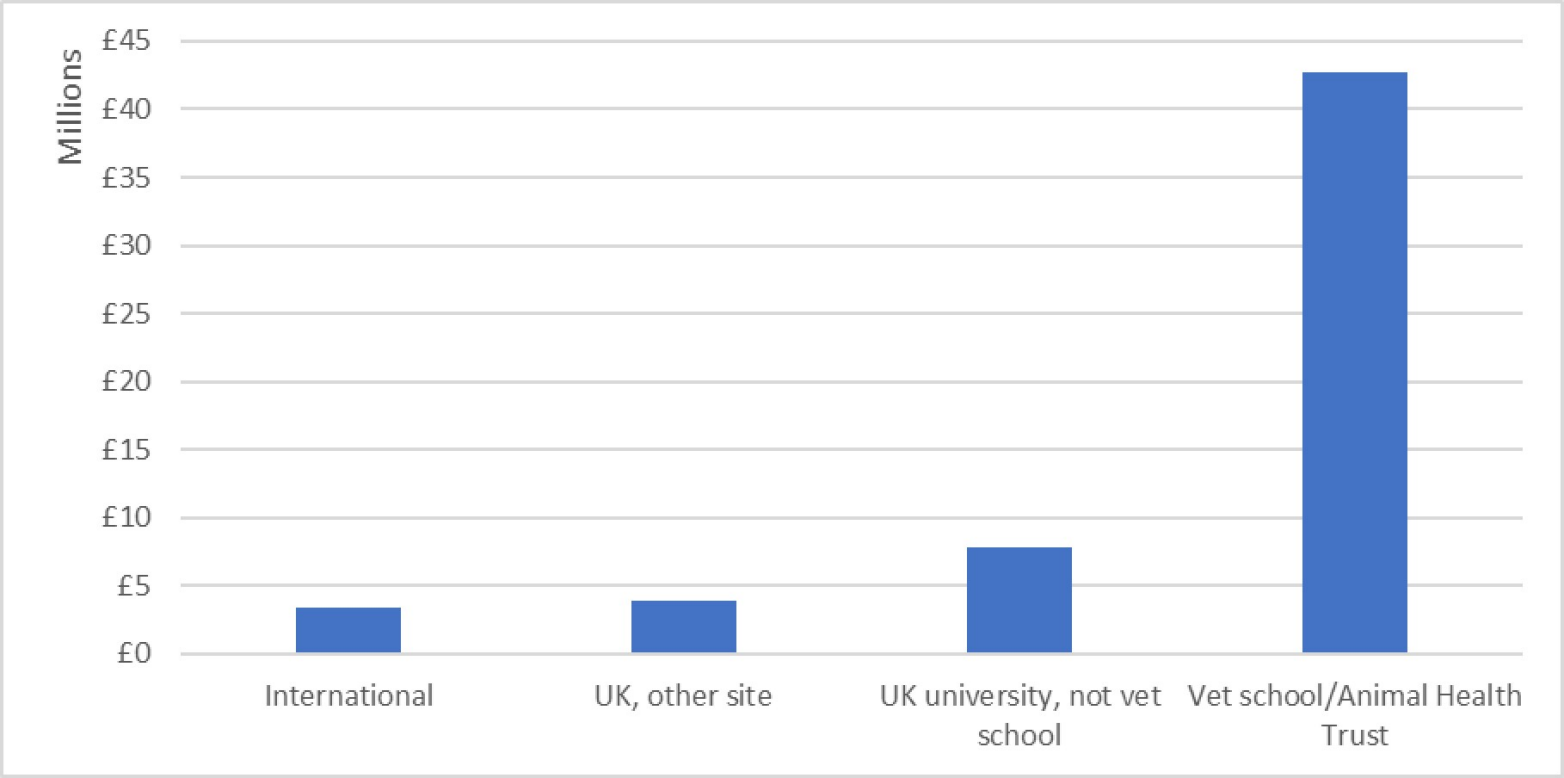
Total UK canine-relevant funding, 2012–2022, categorised by type of main recipient institution.

As previously described, each in-scope research grant was assigned to a main recipient institution, and these were grouped into four categories: UK universities with a veterinary school (including the Animal Health Trust (AHT)), UK universities without a veterinary school, other UK destinations, and international institutions of all types (Fig 4). Most international recipient institutions were veterinary or science departments at universities. Together, these non-UK institutions received £3.4 million (6.0%) of research funding. Some grants were awarded to researchers at non-university centres in the UK, such as specialist veterinary practices or private companies (in the case of seed grants from Innovate UK); these institutions received £3.8 million (6.7%) of in-scope funding. However, most funding, £50.5 million (87.3%), was awarded to UK universities, which were further divided into two groups: those that included a veterinary school and those that did not. UK universities with a veterinary school received £42.7 million (73.8%) of UK canine-relevant funding, while UK universities without a veterinary school received £7.8 million (13.6%) of such funding. The great majority of canine-relevant UK research funding was thus directed to UK veterinary schools or other departments at universities with veterinary schools.

**Fig 5:**
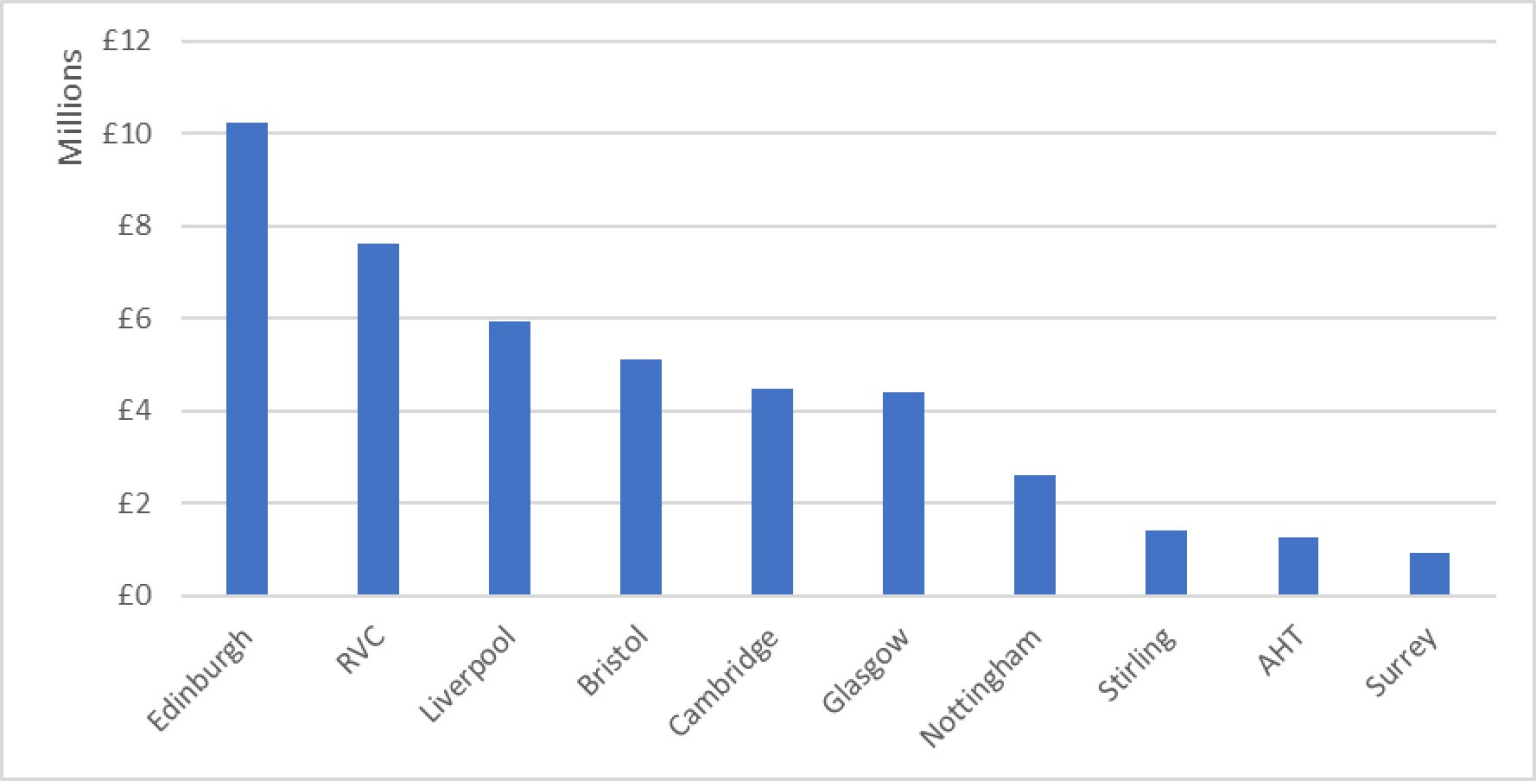
Top ten UK universities by total in-scope canine relevant research funding, 2012–2022. (RVC = Royal Veterinary College, AHT = Animal Health Trust).

To investigate this further, the top ten UK institutions by total in-scope canine-relevant research funding were identified, as shown in Fig 5. Eight of these institutions have a veterinary school. The exceptions are the Animal Health Trust, which was discussed previously, and the University of Stirling, which, although it has hosted other canine-relevant research projects, appears on this list because of a single UKRI grant for > £1 million that investigated cognition across species. However, funding was not evenly distributed between these institutions. Edinburgh, the top-placed university for funding in this study, received more than ten times as much canine-relevant funding (£10.3 million) as the tenth-placed University of Surrey (£0.9 million), a newcomer to the sector which only opened its veterinary school in 2014.

**Fig 6:**
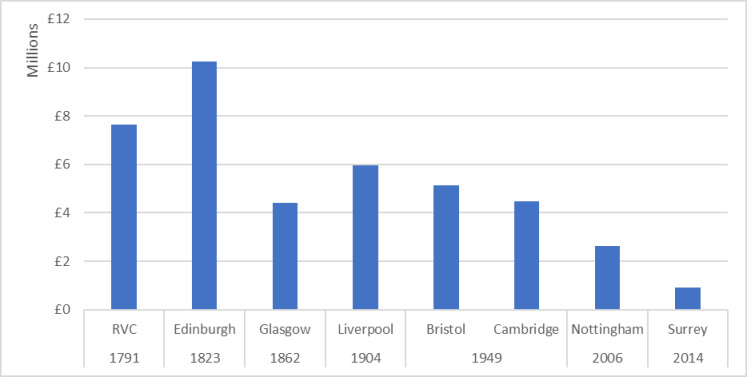
Canine-relevant funding 2012–2022 by UK veterinary school, arranged by date of foundation.

This discrepancy highlights another underlying pattern to this data: universities with older centres of veterinary expertise attracted more canine-relevant funding, as shown in Fig 6. Edinburgh was particularly high performing because of the Roslin Institute’s £3.7 million Ensembl project funding; as previously discussed, this is a multi-species livestock genomics project with a subsidiary canine element. The veterinary schools at Nottingham and Surrey are relative newcomers, founded in the 21^st^ century, which have implemented a hub-and-spoke model that devolves much clinical teaching to satellite veterinary practices, rather than running a centralised clinical teaching hospital; this may also impact their ability to attract clinical research funding. Consequently, Surrey’s canine-relevant research exemplifies the significance of individual researchers in raising the research profile of newer institutions. This is discussed further in a detailed supplementary case study derived from the project data (see S1 Appendix).

### 3.6 Analysis of key research topics

As previously described, eight topics were chosen for this element of the analysis, selected for their broad relevance across the canine health sector. These were:

1. Antimicrobial resistance (AMR)
2. Breed-related disease (BRD)
3. Conformation-related disease (CRD)
4. Canine genetics
5. Neoplasia
6. Clinical relevance
7. Human-animal interactions (HAI)
8. Canine behaviour

The detailed comparative analysis of these topics across funding organisations and sectors is provided in the linked spreadsheet dataset (S1 Table). Breed community funding is subsumed within other animal-directed funding.

#### 3.6.1 Antimicrobial resistance

Within the current dataset, 57 (9.4%) of the 608 (GADAG collapsed) grants investigated various aspects of AMR, ranging from epidemiological surveillance of pathogens in particular canine populations to the speculative development of new antibacterial technologies. In total, £6.4 million (11.0% of total funding) was directed towards these projects, with £4.1 million (7.1% of overall funding and 64.4% of total AMR funding) provided by wide-scope funders (n = 10 grants, including three no-allocated-cost PhD projects) and £2.3 million (3.9% of overall funding and 35.6% of total AMR funding) provided by animal-directed funders (n = 47 grants). Wide-scope funders and animal-directed funders did not differ significantly in the proportional number of AMR research grants they provided (*X*^2^ = 1.377, df = 1, *p* = 0.241). However, AMR-related grants provided by wide-scope funders tended to be much larger than those provided by animal-directed funders, with a median grant size of £505K (IQR £305K - £731K, range £102K - £1.4 million) for wide-scope funders (n = 7 grants, excluding three no-allocated-cost PhD projects), fifty times greater than the median AMR-related grant size of £10K (IQR £5K - £36K, range £1K - £510K) for animal-directed funders (n = 47 grants).

At the level of individual funders, AMR funding was not evenly distributed. Among wide-scope funders, only MRC, ESRC and BBSRC funded any AMR research, yet this topic accounted for 23.2% of all MRC funding in this dataset (£1.1 million) and 64.4.% of all ESRC funding in this dataset (£2.2 million). Animal-directed funders which were less likely to support AMR research included breed communities (which, unsurprisingly, did not fund any) and the KCCT, which only funded one AMR grant. However, Dogs Trust (£1.5 million, 21.2% of all their funding), PetPlan Charitable Trust (£505K, 18.0% of funding) and BSAVA PetSavers (£180K, 19.2% of funding) all supported this topic strongly.

#### 3.6.2 Breed related disease (BRD) and 3.6.3 conformation-related disease (CRD)

Within the current dataset, 222 (36.5%) of 608 (GADAG collapsed) grants were at least partly concerned with all types of heritable BRD, including conformation-related disease, monogenic disorders and polygenic or multifactorial inherited traits. Overall, BRD attracted funding of £18.2 million (all GADAG funding included), 31.5% of all funding, with £10.0 million (17.4% of overall funding and 55.2% of BRD funding) provided by wide-scope funders (n = 29 grants) and £8.2 million (14.1% of overall funding and 44.8% of BRD funding) provided by animal-directed funders (n = 193 grants, GADAG collapsed). Across the entire dataset, animal-directed funders were more likely to fund BRD research than wide-scope funders (*X*^2^ = 22.30, df = 1, *p*<0.001), allotting approximately twice the proportion of their overall funding to this topic (BRD work received 42.7% of grants and 49.0% of funding from animal-directed funders, compared to 20.0% of grants and 24.4% of funding from wide-scope funders). At the individual funder level, there was considerable variation in BRD support. In financial terms, the biggest contributors were BBSRC (£6.9 million), KCCT (£3.3 million), Dogs Trust (£2.5 million), MRC (£1.5 million) and Wellcome Trust (£1.5 million): KCCT, UFAW and breed communities all spent more than 2/3 of their total funds on BRD research (100% for breed communities). Other wide-scope funders, apart from NC3Rs, funded no BRD research, but most animal-directed funders provided at least some support for this topic, reflecting its substantial significance in this sector.

This does not mean, however, that all types of breed-related disease were supported equally by all these organisations. Using the previously described definition of conformation-related disease, 61 grants (10.0% of the total) in the overall (GADAG collapsed) data set were at least partly concerned with CRD, including epidemiological surveys of breed health that included CRD alongside other problems. The total funding for CRD was £4.2 million, 7.16% of all funding, with £226K (0.39% of all funding and 5.5% of CRD funding) provided by wide-scope funders (n = 2 grants) and £3.9 million (6.7% of overall funding and 94.5% of CRD funding) provided by animal-directed funders (n = 59 grants, GADAG collapsed). CRD received 1.38% of total grants and 0.55% of total funding from wide-scope funders, compared to 12.7% of grants and 23.5% of funding from animal-directed funders. Animal-directed funders were thus significantly more likely to fund CRD research than wide-scope funders (*X*^2^ = 15.78, df = 1, p <0.001), offering 17x more financial support for this particular topic, despite only contributing 1/3 of total funding in the overall dataset.

Moreover, both within and between sectors, CRD research funding was very unevenly distributed between funding bodies. The biggest financial contributors were KCCT (£2.4 million) and Dogs Trust (£1.1 million): these two canine-specific charities together provided 83.2% of all CRD funding (£3.4 million), with 57.5% provided by KCCT alone and comprising 60.2% of total KCCT funds. Breed communities supported CRD relatively less, with only eight breed community grants directed to this topic, comprising 21.8% of their total funding, despite 100% of their funding being directed towards BRD more broadly.

#### 3.6.3 Canine genetics

In the overall (GADAG collapsed) dataset, 177/608 (29.1%) of grants supported research that at least partly investigated some aspect of canine genetics (mostly, but not exclusively, concerning molecular genetics). Altogether (all GADAG funding included), this funding totalled £22.7 million, 39.3% of total funding, with £16.0 million (27.6% of overall funding and 70.3% of canine genetics funding) provided by wide-scope funders (n = 41 grants) and £6.8 million (11.7% of overall funding and 29.7% of canine genetics funding) provided by animal-directed funders (n = 136 grants, GADAG collapsed). Canine genetics research received 28.3% of all grants and 38.8% of all funding from wide-scope funders, compared to 29.4% of grants and 40.7% of funding from animal-directed funders. Across the entire dataset (n = 608), proportional contributions to canine genetic research did not differ significantly between wide-scope and animal-directed funders (*X*^2^ = 0.064, df = 1, *p* = 0.800).

Among individual funders, seven organisations, from both wide-scope and animal-directed sectors, provided > £1 million to research projects related to canine genetics: BBSRC (£10.5 million, 55.6% of their total funding); Wellcome Trust (£2.7 million, 36.7% of their total funding); Dogs Trust (£2.4 million, 35.0% of their total funding); KCCT (£2.0 million, 51.6% of their total funding); Pet Plan Charitable Trust (£1.8 million, 62.2% of their total funding); MRC (£1.5 million, 31.4% of total funding); and NERC (£1.2 million, 34.5% of total funding), together addressing a vast range of topics. Despite their smaller market share, breed communities directed a greater share of their total funding to canine genetics research than any other funder (70% of their grants and 74.3% of their funding), reflecting their focus on inherited disease. Among other animal-directed funders, there were some unexpected variations. For instance, BSAVA PetSavers (11.3% of grants, 10.2% of funding) and Waltham (14.3% of grants, 11.3% of funding) proportionately fund much less research linked to canine genetics than PetPlan Charitable Trust (41.4% of grants, 62.2% of funding).

#### 3.6.4 Neoplasia

Within the current overall dataset, 80/608 grants supported research that was at least partly concerned with investigating neoplasia. These projects investigated cancer genetics and genomics, researched new cancer biomarkers and treatments, and tracked the demographic epidemiology of canine cancers. Altogether (all GADAG funding included), this funding totalled £6.7 million, 11.5% of total funding, with £3.2 million (5.4% of overall funding and 47.3% of neoplasia funding) provided by wide-scope funders (n = 8 grants) and £3.5 million (6.1% of overall funding and 52.7% of neoplasia funding) provided by animal-directed funders (n = 72 grants, GADAG collapsed). Neoplasia research received 5.5% of all grants and 7.6% of all funding from wide-scope funders, compared to 15.5% of grants and 21.1% of funding from animal-directed funders. However, across the entire dataset (n = 608), there was no significant proportional difference between the numbers of neoplasia grants provided by wide-scope and animal-directed funders (*X*^2^ = 9.73, df = 1, *p* = 0.002).

Among individual funders, only three organisations provided > £1 million of total funding to support projects that at least partly investigated neoplasia: KCCT (£1.3 million, 31.7% of their funding); Wellcome Trust (£1.2 million, 16.13% of funding) and BBSRC (£1.2 million, 6.1% of funding). Other significant contributors, donating > £500K to this work, were PetPlan (£879K, 31.3% of funding), Dogs Trust (£870K, 12.5% of funding) and NERC (£730K, 21.8% of funding). Innovate UK was the only other wide-scope funder to support neoplasia research at all, with a single grant worth £99K. However, almost all animal-directed funders provided at least some support for neoplasia research, the exceptions being Battersea (a newcomer with only two grants in this dataset) and SCAS (for whom it was out of scope).

#### 3.6.5 Clinical relevance

Using the previously described definition, in the overall (GADAG collapsed) dataset, 326/608 (53.6%) of grants supported clinically relevant research. Altogether, this funding totalled £17.8 million, 30.8% of total funding, with £8.5 million (14.7% of overall funding and 47.7% of clinically relevant funding) provided by wide-scope funders (n = 23 grants, including three no-allocated-cost PhD projects) and £9.3 million (16.1% of overall funding and 52.3% of clinically relevant funding) provided by animal-directed funders (n = 303 grants, GADAG collapsed). Clinically relevant research received 15.9% of all grants and 20.6% of all funding from wide-scope funders, compared to 21.1% of all grants and 65.4% of all funding from animal-directed funders. Across the entire dataset (n = 608), animal-directed funders were significantly more likely to fund clinically relevant research than wide-scope funders (*X*^2^ = 109.12, df = 1, *p*<0.001). However, the £10K median grant size from animal-directed funders (IQR £4K - £25K, range £300 - £538K, n = 303) was thirty times smaller than the median £304K grant size from wide-scope funders (IQR £101K - £451K, range £22K - £2.29 million, n = 20, excluding three no-allocated-cost PhD projects).

Among individual organisations, six funders, divided across both wide-scope and animal-directed sectors, provided > £1 million of total funding for clinically relevant research. These were: Wellcome Trust (£3.5 million, 48.9% of their funding); Dogs Trust (£3.2 million, 45.9% of their funding); MRC (£2.7 million, 55.7% of their funding); Pet Plan Charitable Trust (£2.3 million, 82.9% of their funding); KCCT (£2.1 million, 51.9% of their funding); and BBSRC (£1.8 million, 9.8% of their funding). For most of these funders, clinically relevant research thus represented a significant proportion of their funding. BSAVA PetSavers was the funder that gave the greatest proportion of its resources to support clinically relevant research, directing 85.6% of its grants and 91.5% of its funding (£861K) towards this area. Moreover, all individually listed animal-directed funders in the dataset, except Battersea and SCAS, provided at least some support for this topic.

#### 3.6.6 Human-animal interactions (HAI)

Over the whole (GADAG collapsed) dataset, 129/608 (21.2%) of grants supported HAI investigations, totalling £12.75 million, 22.06% of total funding. Wide-scope funders provided £9.4 million (16.2% of overall funding and 73.4% of HAI funding, n = 40 grants) of funding, and animal-directed funders provided £3.4 million (5.9% of overall funding and 26.6% of HAI funding, n = 89 grants, GADAG collapsed). HAI research received 27.6% of all grants and 22.7% of all funding from wide-scope funders, compared to 40.7% of all grants and 20.44% of all funding from animal-directed funders. The proportional number of grants allotted to HAI research by wide-scope funders was significantly greater than that provided by animal-directed funders (*X*^2^ = 4.62, df = 1, *p* = 0.032).

Individual organisations varied greatly in their support for HAI research. Among individual organisations, four wide-scope funders and one animal-directed funder provided > £1 million of total funding for HAI research. These were: Wellcome Trust (£3.8 million, 52.6% of their canine-relevant funding); AHRC (£1.5 million, 100% of their funding); NERC (£1.2 million, 34.5% of their funding); UKRI (£1.0 million, 49.5% of their funding) and Dogs Trust (£2.4 million, 34.0% of their funding). Besides AHRC, three other organisations directed 100% of their in-scope funding towards HAI research: ESRC; Battersea and SCAS. Wide-scope funders which supported HAI research funded humanities or social science studies that investigated various aspects of the human-canine relationship from an abstract or academic perspective, while animal-directed funders such as Battersea and SCAS both funded more applied studies that looked at specific aspects of human-canine interactions. In contrast, some organisations directed no funding towards HAI studies, including MRC, EPSRC, PetPlan Charitable Trust and breed communities.

#### 3.6.7 Practical canine behaviour

Over the whole (GADAG collapsed) dataset, 61/608 (10.0%) of grants supported dog-facing behavioural investigations, totalling £4.9 million, 8.4% of total funding. This could be divided into £2.6 million provided by wide-scope funders (n = 14 grants; 4.4% of overall funding and 52.6% of canine behavioural funding) and £2.3 million provided by animal-directed funders (n = 47 grants; 4.0% of overall funding and 47.4% of canine behavioural funding). Practical canine behavioural research received 9.7% of all grants and 6.2% of all funding from wide-scope funders, compared to 10.2% of all grants and 13.8% of all funding from animal-directed funders. Two funders allotted more than £1 million to research that concerned canine behaviour: the wide-scope funder BBSRC (£1.8 million, 9.75% of its funding) and the animal-directed funder Dogs Trust (£1.7 million, 24.9% of its funding).There was no significant difference between the proportional number of grants allotted to this research by wide-scope and animal-directed funders (*X*^2^ = 0.352, df = 1, *p* = 0.553).

However, although the wide-scope and animal-directed sectors overall contributed roughly equal amounts of funding to canine behavioural research, the distribution of support between funders differed widely between these sectors. Only three wide-scope funders supported this topic at all, engaging with very different fields of investigation: NC3Rs supported work to improve the wellbeing of laboratory dogs; Innovate UK supported the development of monitoring technology; and BBSRC mostly supported research into canine brain health and behaviour. In contrast, all animal-directed funders except PetPlan Charitable Trust and SCAS (which focused exclusively on the human side of HAI) directed at least some support to dog-facing studies of canine behaviour and cognition, with the 47 grants in this sector covering a wide range of approaches to advance canine mental wellbeing.

### 3.7 Research impact metric analysis: ‘Benefit for the dog’ and ‘pathway to impact’

#### 3.7.1 ‘Benefit for the dog’ (BFD)

Across the whole dataset of 608 grants (GADAG collapsed), BFD scores ranged from 1–25 (with some prime number gaps resulting from the multiplication methodology); the possible maximum was 30. No grants were rated as potentially changing the outcome of a usually/always fatal disease for millions of dogs (i.e., there were none that scored 30). This was because rabies, the only featured generally fatal disease to affect large numbers of dogs, is (despite its impact on affected dogs and its zoonotic significance) undoubtedly much less prevalent as a clinical disease in the global canine population than, for example, helminthiasis (parasitic worms), so was not assigned the highest possible score [38].

**Fig 7:**
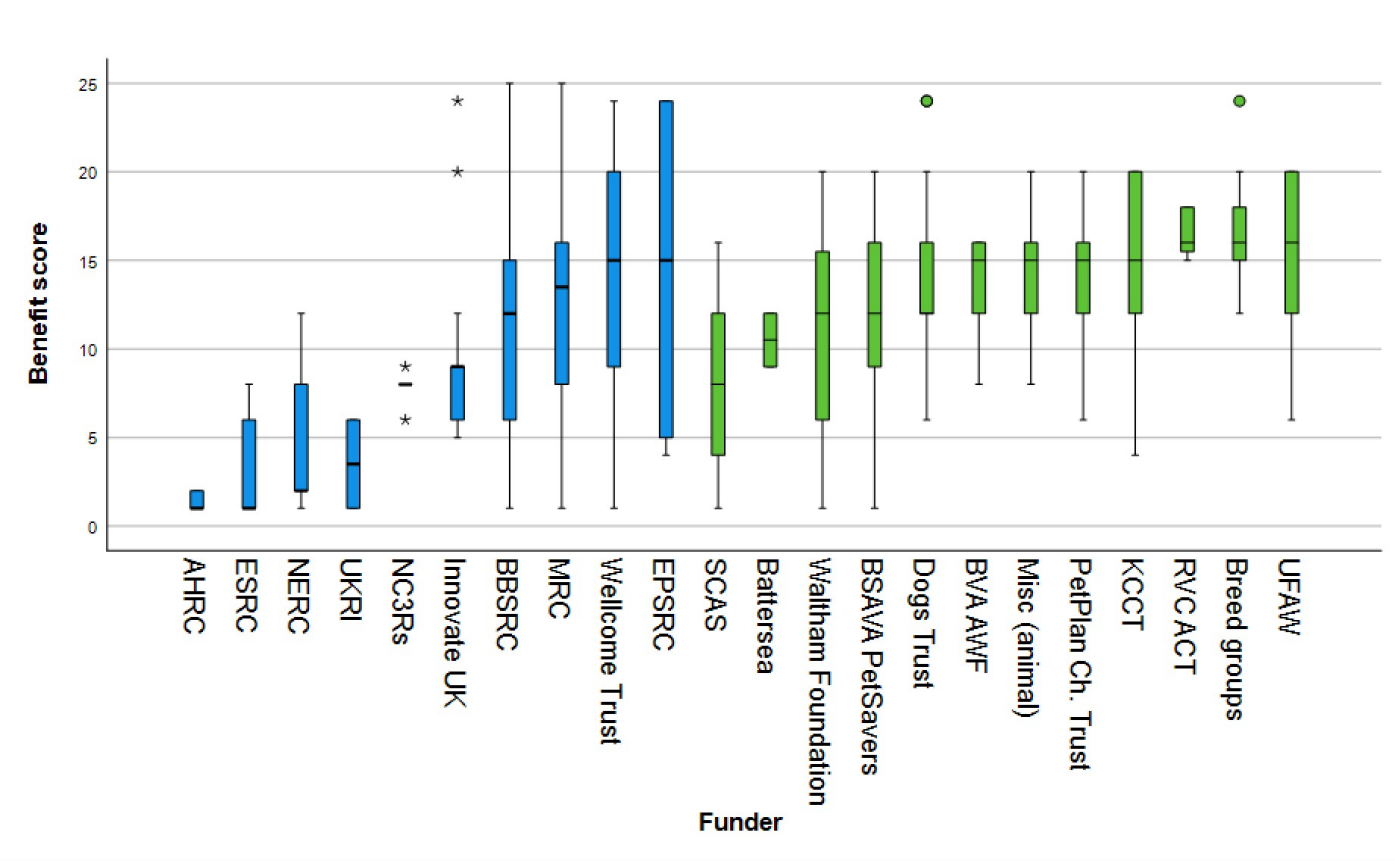
Box-and-whisker plot showing benefit for dog score for UK canine-relevant research funding, categorised by funder and ordered by ascending median within wide-scope funder group (blue) and animal-directed funder group (green), 2012–2022.

For the overall dataset, the median BFD score was 15 (IQR 9–16, range 1–25). Dividing the data by funding sector, the median BFD score for wide-scope funders (n = 145 grants) was 9 (IQR 6–15, range 1–25). The median BFD score for animal-directed funders (n = 463 grants, breed communities included, with GADAG collapsed) was 15 (IQR 12–16, range 1–24). Overall, BFD scores for grants from animal-directed funders were significantly higher than those for wide-scope funders (Mann-Whitney U = 45235, p<0.001). Fig 7 shows a box and whisker chart for all main funders, grouped by funding sector.

To investigate BFD data further, the lowest (BFD <9) and highest (BFD >16) quartiles of the overall data were explored in more detail. Across the whole dataset, 120 grants were assigned a BFD score of <9. These included 65 grants from wide-scope funders (44.8% of all grants from this sector) and 55 grants from animal-directed funders (11.9% of all grants from this sector). Grants provided by wide-scope funders were thus about four times more likely to have a BFD score in the lowest quartile than those provided by animal-directed funders, reflecting their less canine-focused portfolios. Across the whole dataset, 89 grants were assigned a BFD score >16. This included 19 grants issued by wide-scope funders (13% of all grants from this sector) and 70 grants issued by animal-directed funders (15% of all grants from this sector). Thus, there was little difference in the percentage of high BFD scoring grants between wide-scope and animal-directed funding sectors.

Individual funders were scrutinised to assess their low and high scoring BFD profiles. Funders that contributed fewer than ten individual canine-relevant grants to the dataset were excluded from this process, as their contributions were potentially skewed by small sample sizes. The remaining funders comprised four wide-scope and eight animal-directed funding organisations or categories, as shown in Table 8.

**Table 8:** Low and high-scoring ‘benefit for the dog’ (BFD) profiles for UK funders of canine health and welfare research, 2012–2022 (>10 grants only, divided by wide-scope and animal-directed sectors).

Funder (>10 grants only)	No of grants	Median BFD**	No with BFD<9	%BFD<9	No with BFD>16	%BFD>16
**Wide-scope**						
BBSRC	66	12	23	34.8%	7	10.6%
Innovate UK	21	9	9	42.9%	2	9.5%
MRC	10	13.5	3	30.0%	2	20.0%
Wellcome	17	15	4	23.5%	6	35.3%
**Animal-directed**						
Breed groups	47*	16	0	0.0%	12	25.5%
BSAVA PetSavers	97	12	20	20.6%	14	14.4%
Dogs Trust	81	12	8	9.9%	11	13.6%
KCCT	51	15	1	2.0%	14	27.5%
PetPlan Charitable Trust	87	15	5	5.7%	7	8.0%
SCAS	19	8	10	52.6%	0	0.0%
UFAW	22	16	1	4.5%	6	27.3%
Waltham Foundation	28	12	8	28.6%	2	7.1%

*Give a Dog a Genome collapsed

**BFD = benefit for the dog.

As previously reported, wide-scope funders consistently supported a high proportion of low-BFD scoring projects; for all four listed wide-scope funders, over 20% of their total grants had a BFD score <9. Such grants concerned abstract, theoretical or lab-based research with little or no direct relevance to canine health and welfare. For animal-directed funders, a more variable proportion of projects were low-scoring, ranging from 0% for breed groups to over 50% for SCAS (Society for Companion Animal Studies); these low-scoring projects concerned a wide range of topics, such as laboratory investigations of cellular processes, audits of veterinary workplace practices and studies of human benefits from pet ownership.

High BFD scoring projects from wide-scope funders invariably dealt with serious diseases. The majority (16/19 projects) concerned infectious diseases, mostly zoonotic. These included rabies (nine projects) and other viral, bacterial and parasitic infections. Most were interdisciplinary epidemiological studies or projects to improve vaccine technology, and the majority adopted a One Health perspective (12/19 projects). Three high-scoring projects from wide-scope funders were coded as canine-focused. All three were BBSRC-funded and concerned the investigation and treatment of canine cancer, in every case framing this as also advancing understanding of an equivalent human disease. High-scoring projects from wide-scope funders thus invariably engaged with canine health and welfare as part of a broader context of investigating significant disease in people or other economically important species.

High BFD scoring projects supported by animal-directed funders had a much broader range of scope. A few of the 70 grants in this category supported One Health investigations into zoonotic diseases such as rabies, like those supported by wide-scope funders. However, 60/70 (86%) of high-scoring grants from animal-directed funders were canine-focused. The majority (48/70, 69%) dealt with a wide range of breed-related diseases; these investigations took many forms, ranging from epidemiological studies and genomic investigations to clinical studies. Many of these grants scored particularly highly because they addressed high-welfare problems that were common within relatively popular breeds, such as BOAS in brachycephalic dogs or histiocytic sarcoma in flat-coated retrievers. Breed-related disease research was discussed in more detail previously. Other high-scoring projects from animal-directed funders tended to target problems that were very serious for affected dogs (such as Alabama rot) and/or widespread within the UK canine sector (such as vaccination uptake).

#### 3.7.2 ‘Pathway to Impact’ (PTI)

**Fig 8:**
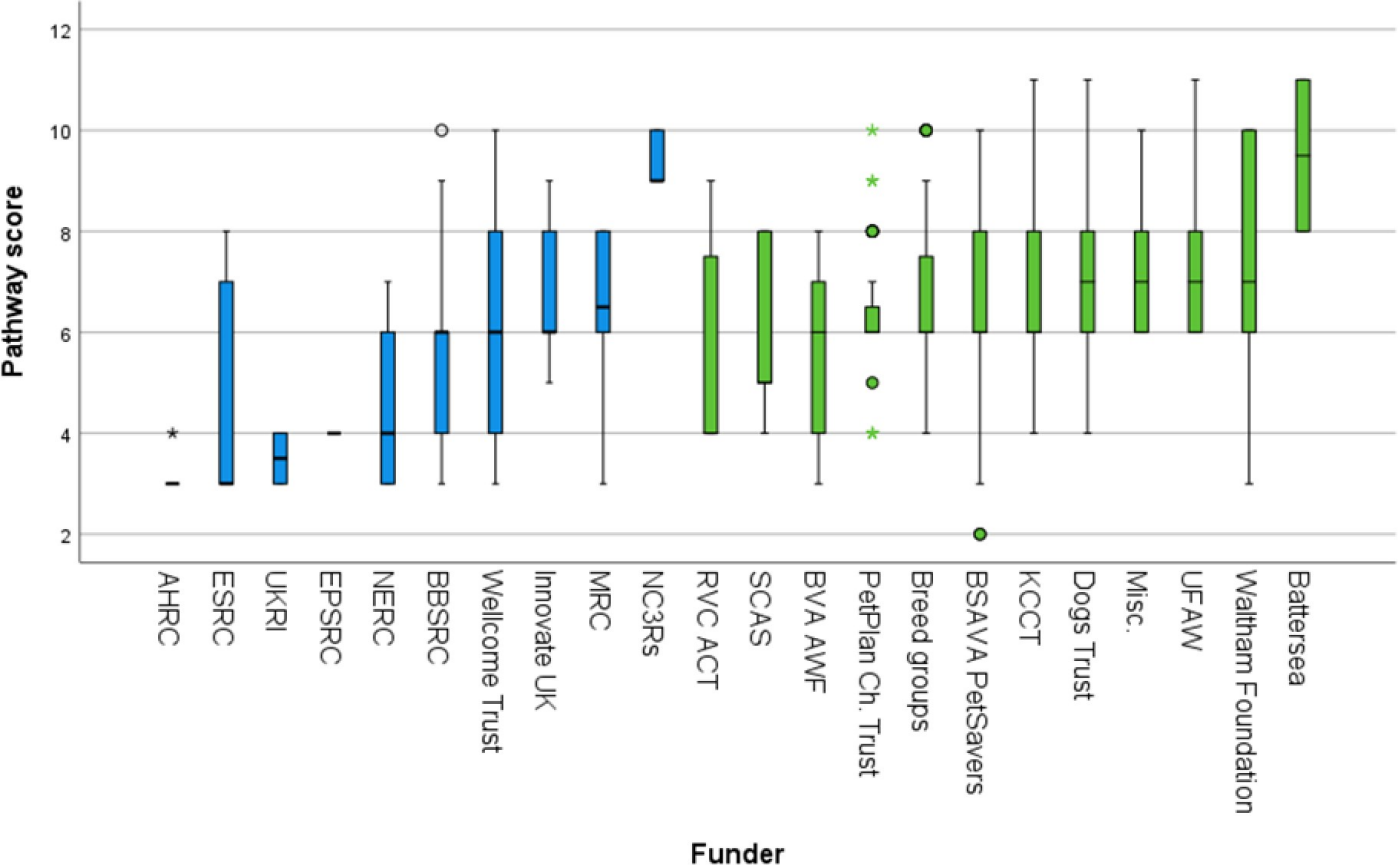
Box-and-whisker plot showing pathway to impact score for UK canine-relevant research funding, categorised by funder and organised by ascending median within wide-scope funder group (blue) and animal-directed funder group (green), 2012–2022.

Across the whole dataset of 608 grants (GADAG collapsed), the median PTI score was 6 from a possible maximum of 11 (IQR 6–8, range 2–11). When considered by funding sector, the median PTI score for wide-scope funders (n = 145 grants) was 6 (IQR 4–6, range 3–10), and the median PTI score for animal-directed funders (n = 463 grants, breed communities included, with GADAG collapsed) was also 6 (IQR 6–8, range 2–11). Overall, however, PTI scores for grants from animal-directed funders were significantly higher than those for wide-scope funders (*p*<0.001, Mann-Whitney U = 43506.5). Fig 8 shows a box and whisker chart for all main funders, grouped by funding sector.

As with the BFD scores, the lowest (<6) and highest (>8) quartile of overall PTI scores were explored in more detail. Over the whole dataset, 118 grants were assigned a low PTI score of <6. Dividing the data by funding sector, 54 grants from wide-scope funders (37.2% of all grants from this sector) were assigned a PTI score <6, and 64 grants from animal-directed funders (13.8% of all grants from this sector) were assigned a PTI score <6. Grants provided by wide-scope funders were thus almost three times more likely to have a PTI score in the lowest quartile than those provided by animal-directed funders, again reflecting their less canine-focused portfolios. Across the whole dataset, 97 grants were assigned a high PTI score >8. Dividing the data by funding sector, 18 grants from wide-scope funders (12.4% of all grants from this sector) were assigned a PTI score >8, and 79 grants from animal-directed funders (17.1% of all grants from this sector) were assigned a PTI score >8.

Finally, individual funders were scrutinised to assess their low and high scoring PTI profiles, using the same process as described for BFD scoring. The same four wide-scope and eight animal-directed funding organisations or categories were selected, as shown in Table 9. The median PTI was calculated for each funder. Using the previously discussed low and high quartile boundaries of 6 and 8 as cutoff points, the number of low-scoring and high scoring grants for each funder was calculated and expressed as a percentage of the total number of grants provided by that funder.

**Table 9:** Low and high-scoring ‘pathway to impact’ (PTI) profiles for UK funders of canine health and welfare research, 2012–2022 (> 10 grants only, divided by wide-scope and animal-directed sectors).

Funder (>10 grants only)	No of grants	Median PTI**score	No. with PTI<6	%PTI<6	No. with PTI>8	%PTI>8
**Wide-scope funders**						
BBSRC	66	6	22	33.33%	5	7.58%
Innovate UK	21	6	3	14.29%	4	19.05%
MRC	10	6.5	2	20.00%	0	0.00%
Wellcome	17	6	7	41.18%	3	17.65%
**Animal-directed funders**						
Breed groups	47*	6	1	2.13%	11	23.40%
BSAVA PetSavers	97	6	22	22.68%	18	18.56%
Dogs Trust	81	7	6	7.41%	17	20.99%
KCCT	51	6	3	5.88%	8	15.69%
PetPlan Charitable Trust	87	6	7	8.05%	6	6.90%
SCAS	19	5	10	52.63%	0	0.00%
UFAW	22	7	0	0.00%	4	18.18%
Waltham Foundation	28	7	6	21.43%	10	35.71%

*Give a Dog a Genome collapsed

** PTI = pathway to impact.

Median PTI scores clustered heavily around the mid-range values of 5–7, reflecting the additive scale and the relative infrequency of extreme overall scores for any funder. Grants awarded by wide-scope funders that received low PTI scores either concerned laboratory-based research with little direct route to impacting canine health or welfare, dealt with very theoretical research questions, or considered dogs within a wider One Health context, so that dogs were not centred in intended research outputs. Most animal-directed organisations funded a smaller proportion of low-scoring PTI projects than wide-scope funders, reflecting their greater focus on canine health and welfare. However, animal-directed funders varied considerably in their proportion of low-scoring grants. Low-scoring grants from animal-directed funders mostly fell into two categories. The majority were laboratory-based investigations that were not intended to produce a direct intervention to improve canine health, but were expected to produce technical behavioural change, at some remove from clinical impact (for example, studying the production or detection of new potential biomarkers for disease). Others studied the human-animal relationship primarily from the perspective of human benefit; these achieved lower scores because any resulting impact for dogs was likely to be delayed or indirect.

There was considerable variation across high PTI scoring projects among both wide-scope and animal-directed funders. Projects that scored highly were generally designed to produce outputs with direct, immediate real-world relevance, which could be implemented without undue difficulty to improve canine health or welfare. These clear pathways to impact took many forms. Examples include: the development of DNA tests for breed-specific monogenic inherited diseases, funded by breed communities desperate to utilise a successful test; clinical trials to validate a new medical or surgical treatment protocol, where clinicians would readily adopt a new approach with a strong evidence base; developing new laboratory techniques to replace the use of dogs for scientific procedures, which researchers would implement willingly; or behavioural studies to better understand shelter dog welfare, funded by organisations such as Dogs Trust that have the agency to then promptly enact any recommendations themselves.

## 4. Discussion

This research represented a novel initiative intended to investigate not-for-profit funding of canine health and welfare research in the UK between 2012 and 2022, a topic that had not previously been studied in detail. It deployed a wide range of exploratory and investigative approaches, with data analyses involving both quantitative and qualitative techniques. The latter were dynamically customised in response to the unique challenges of this dataset and the aims of this study. Some aspects of the methodology were modified iteratively during the investigation, and, as noted above, the exact boundaries of the dataset were inevitably imprecise, so that the results should be taken as indicative rather than firmly definitive. As previously described, not all in-scope data could be harvested. Moreover, not all scoping decisions were clearcut, either on an organisational level or on a grant level. For example, research funded by corporate practices was excluded, although some corporates now offer some research grants that are not limited to internal candidates, because much corporate research funding is still currently offered internally or with a stated preference for internal research partnership. Conversely, UKRI funding was deemed canine-relevant even if it concerned a topic such as archaeology with less direct impact on canine health and welfare, on the basis that such information does contribute to understandings of canine coexistence with humans. Furthermore, despite efforts to standardise the processes, some individual grant inclusion decisions inevitably remained somewhat subjective, contributing another element of imprecision to the dataset boundaries. Therefore, it is likely that another lead researcher would have created a dataset with some minor differences in the selected entries; however, given the large sample size (n = 684 grants) and sustained attempts to define and standardise assessment processes, the impact on overall results should be minimal.

Similarly, some aspects of the QCA coding process developed and used in the current analysis reflect the approaches of the current researchers and could have been deployed differently by another research team. Since a similar dataset had not been coded previously, the coding frame for this project was developed *ab initio*, with minimal scaffolding from previous investigations, so that the current analytical process evolved iteratively. This meant that not all aspects of primary coding were equally productive. For example, in the primary coding phase, all research grants were coded according to twelve dimensions, as shown in Table 1. However, not all dimensions were used in subsequent analysis, because some (such as ‘specific knowledge field’ or ‘project output’) later proved to offer more useful insight than others (such as ‘human role in study’ or ‘research approach’).

The ‘interpretive impact’ metrics of ‘benefit for the dog’ (BFD) and ‘pathway to impact’ (PTI) were included at the request of the project funders, for whom a better understanding of these parameters was a key desired project outcome. However, these metrics are inherently highly subjective, and could have been represented in various ways. Developing a scoring framework to determine the potential value of a research project in advancing canine health or welfare (BFD) offered several challenges. Firstly, it is difficult to compare the welfare impact of entirely different types of problem, such as puppy smuggling, atopy and lymphoma. Secondly, the overall score had to encompass both the severity and duration of the problem addressed; but a long-lasting condition of moderate severity (e.g., periodontal disease) might reduce lifetime wellbeing far more than a short-lived but more severe episode of illness (e.g., gastric torsion). Thirdly, as discussed previously, the eventual metric also had to consider the worldwide numbers of dogs both affected by the problem and likely to benefit from any intervention resulting from the research.

The final BFD metric attempted to balance these variables as far as possible. It did not assess the actual implementation of any potential benefits, which was considered under pathway to impact. Nor did it attempt to measure the scientific quality or credibility of the research proposal, or the effectiveness of the proposed output in solving or mitigating the problem addressed by the research question. These factors were not considered because, in many cases, there was inadequate information to make such judgements, and because, even where a full project description was available, one person could not scrutinise all projects equally rigorously. This underlines the value of appointing an expert review panel to make informed funding decisions. There were similar difficulties in developing the ‘pathway to impact’ (PTI) score, particularly with negotiating the difference between projects explicitly intended to produce an immediate real-world benefit and those intended as pilot, ‘back office’ or exploratory studies, where direct practical impact was not expected even if the subject matter was directly relevant to canine health or welfare. Consequently, as previously discussed, the eventual metric should be interpreted more as a measure of positionality than of research quality.

The results presented in this paper inevitably represent just one selection of analyses among many possibilities for this dataset. To some extent, these choices were driven by, and emerged from, the underlying patterns inherent in the dataset itself. For example, the breakdown of funding data over time (Fig 1) revealed no underlying temporal trend in funding, largely due to overwhelming ‘noise’ in the data, as previously described, potentially exacerbated by partial data retrieval and incomplete data. Therefore, subsequent data analysis in this paper did not consider changes over time but explored underlying funding patterns in other ways. Consequently, it is possible that other temporal trends went unrecognised. In contrast, comparisons between wide-scope and animal-directed funders provided helpful insight, so were deployed as a major strand of analysis running through this research.

One of the most illuminating dimensions developed in the primary QCA coding was the categorisation of projects according to their broad focus (‘One Health’, canine-focused, etc.). As previously discussed, the predominance of animal-directed funders in canine-focused research reveals the critical importance of these funders within this sector: research that centres the dog largely relies on animal-directed funding. Since such funding is a limited resource, this provides useful evidence to confirm the critical importance of strategically directing such funding as effectively as possible to maximise its value in advancing canine health and welfare. Moreover, this underlines the value of engaging with wide-scope funders where possible, to tap into their far greater resources. While the current study data only concern canine-relevant funding, it is noteworthy that the entire funding landscape described here is very small compared with overall funding of medical research. For context, the 2021/22 MRC Research and Innovation budget alone was £709 million [39], so that a single component of UK medical research funding in a single year was more than tenfold larger than the total £58 million UK canine-relevant funding identified over the whole timespan covered by this paper. The broad-focus analysis in this paper reveals that a substantial proportion of funding from wide-scope providers was allocated to research which, while still canine-relevant, was primarily positioned within a One Health context. The veterinary sector frequently argues that the medical sector overlooks the importance of One Health [40–42]. For these reasons, One Health funding may represent a proportionally more significant opportunity in the canine health and welfare sector than in the far larger world of medical research funding. Researchers in the canine sector who can adopt a One Health perspective may thus find this approach facilitates their access to sizeable funding streams.

Given that research funding is a finite and limited resource, it is concerning that projects supported by wide-scope funders were significantly more likely to lead to published output than those funded by animal-directed organisations. Information bias may have contributed somewhat to this apparent discrepancy, with data available for wide-scope funders being more accurate because online grant data generally included project outputs. In contrast, grant data had to be individually researched for many animal-directed funders, sometimes from very limited available data, so that some public outputs may have been missed. Moreover, not all supported projects were equally likely to, or even intended to, produce a formal productive output. For example, some animal-directed funders, such as BSAVA PetSavers, regularly provide smaller grants to support undergraduate student research projects. Arguably, the main aim of these grants is to facilitate the training of new researchers–a vital part of the research jigsaw in itself–and it is unsurprising that, although BSAVA encourages these studies to be presented at professional conferences, it is less common for them to become peer-reviewed publications. Similarly, some funders, such as Dogs Trust, have a deliberate policy of allocating some funds to relatively small-scale pilot projects. These can produce groundbreaking progress in new fields but may be less likely to succeed than those which explore better-known territory; moreover, some could have led into further successful work which was not transparently linked to the original award. Some grants also represented successive funding contributions to research which is still ongoing; this is particularly true of genetic research into inherited canine disease, which often involves multiple small funding injections to pursue one gene test over many years.

Despite these provisos, however, in most circumstances there is an implicit or explicit expectation that research grants will lead to published results. Yet the sector discrepancy between research output success in this dataset suggests a contrast in funding culture and expectations from grantholders between wide-scope and animal-directed funders. There is also considerable variation in the extent to which funding bodies track grant outputs. Wide-scope funders have formalised processes to mandate reporting of outcomes. For example, the Wellcome Trust requires most grantholders to repeatedly complete a comprehensive report describing all grant outputs during the five years after a grant terminates: failure to comply renders the grantholder ineligible for future grants [43]. In general, animal-directed funders impose less accountability. Some have little or no internal scrutiny of grant outcomes. Some (such as Dogs Trust) do require reports from grantholders. A few, such as BVA AWF, achieve excellent transparency of grant outcomes, with past grants and their outputs listed in detail on the charity’s website [44]. However, such clarity for outputs is not standard. There is also a potential power imbalance when breed communities fund research: here, accountability usually lies with the researcher personally rather than in any formalised contract. Overall, there is clear scope for increased transparency and accountability of grant outputs across the animal-directed funding sector, although it should be noted that the temporal lag in the current analysis means that any recent improvements in these processes would not have been visible in the pre-2018 data considered here.

The breakdown of funding by main recipient institution revealed a strong skew of research funding towards UK universities with a long tradition of veterinary expertise. This was particularly true for the University of Edinburgh, which outperformed even the expectations conferred by its 200 years of history, as shown in Fig 6. As previously discussed, this was largely due to substantial BBRSC funding of the Roslin Institute’s Ensembl project to create an annotated domestic animal reference genome. The Roslin Institute has been part of the Royal (Dick) School of Veterinary Studies since 2008 [45], but the study of genetics in both organisations has roots in Edinburgh’s Department of Zoology as long ago as 1910 [46], so that this leading centre of current expertise has developed during a century of cumulative research knowledge generation across multiple disciplines. Moreover, leading expertise creates a snowball effect, attracting additional support from other funders for further work that draws on a key resource. This exemplifies the complex networks that characterise UK canine-relevant research, where multiple funders may contribute to different stages of linked research initiatives.

More general arguments can be made both for and against the skew of research funding towards institutions which already receive large funding shares. These centres need adequate financial support to remain key players in a global marketplace, and knowledge generation can be stimulated by the synergy of a sizeable research environment that attracts investigators with complementary skills. Yet innovative project ideas can also arise from less high-profile institutions or from researchers with unexpected skill sets, so there may be value in encouraging submissions from research centres that are not currently heavily funded, perhaps by deliberately advertising funding calls in ways that reach researchers in a wider range of institutions. A comparison of successful and unsuccessful grant applications would offer useful insight here, potentially revealing whether larger institutions have more chance of successful funding outcomes. However, most organisations which shared data for the current project were unable or unwilling to provide information on unsuccessful grant bids, because of concerns about data protection regulations and/or lack of relevant records. Therefore, such an analysis was not possible within the current study.

The analysis of eight key research topics by funding sector and individual funder revealed some interesting patterns and surprising focal insights, which are potentially useful to a range of stakeholders. These data enable researchers to assess which funders are more or less likely to support particular types of research. Similarly, the current study data provide an overview of recent sector funding patterns, enabling funding organisations to compare their own priorities with those of others as they consider future funding strategies.

Briefly considering each specific research topic in turn, these data reveal that many funders across both wide-scope and animal-directed sectors, apart from those that have other obvious priorities, are willing to support research into antimicrobial resistance (AMR) and already include it within their portfolios. In contrast, as previously discussed, both breed-related disease (BRD) in general and conformation-related disease (CRD) research, more specifically, are preferentially supported by animal-directed funders, particularly by Dogs Trust and KCCT. Although BBSRC was also a major funder of BRD research, the projects it supported were largely presented in a ‘one medicine’ context and did not concern CRD. Moreover, most breed community funding reported in this dataset did not engage with CRD at all, despite its 100% direction to BRD more broadly. Breed community CRD funding was all provided by breed-specific registered charities concerned with either IVDD in Dachshunds or CM/SM in CKCS [47,48]. It is noteworthy that, with these two exceptions, breed communities only financially supported research into diseases that were not directly related to their own aesthetic preferences, reflecting the political difficulties inherent to this sector.

Canine genetics research received approximately 40% of all funding from both the wide-scope and animal-directed sectors, indicating that such research appears to be a key priority for many funders. However, the detailed data on its funding revealed some variation among animal-directed funders in their support for this topic: this insight could be very helpful to those submitting a funding bid in this field. Research into canine neoplasia also attracted support from both sectors but was prioritised by a greater proportion of animal-directed funders than wide-scope funders, demonstrating the high profile of cancer research across the canine health sector. Similarly, clinically relevant research was more strongly supported by the animal-directed sector in general and by certain particular funders (notably, though unsurprisingly, BSAVA PetSavers), again demonstrating the strategic importance of targeted funding from the animal-directed sector in resourcing advances in canine health.

The differences in broad HAI funding between wide-scope and animal-directed sectors generally reflected their different foci, with wide-scope HAI funding largely supporting more abstract research topics while animal-directed funding was more concerned with applied investigations. It is perhaps surprising that PetPlan Charitable Trust did not fund any HAI research, but it is useful for researchers to be aware that this organisation evidently focuses exclusively on physical disease instead. The limited engagement of wide scope funders with practical canine behavioural research is to be expected, given that this sector seldom supports canine-focused topics. However, most animal-directed funders did support dog-facing investigations of canine behaviour and welfare. This is encouraging information for researchers interested in this field: while animal-directed funders may have smaller budgets than wide-scope organisations, they evidently appreciate the significance of behavioural health to canine welfare and are mostly willing to consider this topic in-scope.

As previously discussed, ‘benefit for the dog’ (BFD) and ‘pathway to impact’ (PTI) metrics were inevitably subjective and required informed interpretation, but nevertheless provided interesting insight, particularly when considering the lowest and highest scoring quartiles. Low BFD scores from wide-scope funders usually indicated research never intended to foreground canine benefit. Low BFD scores among animal-directed funders can be explained in two ways. Some projects, such as most of the low-scoring BSAVA PetSavers grants, were small grants allotted to narrow-focus clinical projects which could not be expected to have a transformative benefit for dogs; for example, audits of routine veterinary workplace practices, which often compared two techniques that were both broadly satisfactory. Others, including all the low-scoring SCAS grants, were studies of the human-animal relationship that were framed to investigate and support the value of animal companionship to humans rather than to explore the impact on the dog. A low BFD score should thus be taken to indicate a project that had alternative priorities, rather than a project that was inherently of low value.

Overall, a large proportion of projects with high BFD scores that were supported by animal-directed funders concerned diseases or problems that did not have a direct connection to human disease, and which therefore would have been less likely to receive support from wide-scope funders. For example, breed groups entirely avoided low BFD scores, because they only funded research which investigated heritable diseases that were already recognised as a significant problem within that breed population. This reiterates the importance of the animal-directed funding sector in supporting research that may have considerable impact on canine lives, but which might otherwise go unfunded.

As with BFD scoring, most low PTI scores reflected studies that were primarily intended to produce outputs that advanced knowledge in other ways, rather than directly improving canine lives. It was seldom possible to identify any significant weaknesses in the design of research projects from the limited information available, and so this was rarely a reason for low scoring.

Moreover, it is important to note that, as previously mentioned, some projects were inherently unable to achieve the highest PTI scores, because of their subject matter. For example, the coding for research which investigated human behavioural issues that impact canine welfare, such as the preference for brachycephalic conformation, or poor purchasing choices among puppy buyers, reflected the political difficulties in implementing any findings in the real world. This does not devalue such work, but rather moderates expectations about what can be achieved. Similarly, many projects investigated ‘back office’ technical procedures, or were intended to explore cutting-edge new concepts, and so were inevitably more removed from front-line impact. This was the case for all MRC funded canine-relevant research, for instance, none of which had a canine-focused perspective.

In summary, deployment of this scoring system was impacted by the varying priorities and scope of different types of research project, and was also inevitably influenced by the subject knowledge of its user/s. Nevertheless, the BTD and PTI tools together provided a novel and helpful way to assess and compare practical relevance to canine health and welfare across the whole broad spectrum of canine-relevant research, and could usefully be developed further in subsequent work.

## 5. Conclusion

This pioneering study offers a novel overview of UK not-for-profit research funding for canine health and welfare from 2012 to 2022. Despite inevitable uncertainties and inaccuracies in the data, the results provide an innovative baseline investigation to inform future funding strategies, and offer insights that are potentially useful to a range of sector stakeholders. A subsequent paper will build on this analysis to explore how this historical funding distribution in canine-relevant research aligns with the current consensus research priorities of sector stakeholders. The resultant synthesis will provide evidence to inform future reform in funding processes and redirection of funding priorities, as appropriate.

## Supporting information

S1 Appendix (DOCX)

S1 Table (XLSX)

## Abbreviations

AHRC: Arts and Humanities Research Council
AMR: antimicrobial resistance
AHT: Animal Health Trust
BBSRC: Biotechnology and Biological Sciences Research Council
BOAS: brachycephalic obstructive airway syndrome
BRD: breed-related disease
BSAVA: British Small Animal Veterinary Association
BVA AWF: British Veterinary Association Animal Welfare Fund
CKCS: Cavalier King Charles Spaniel
CM/SM: Chiari-like malformation/syringomyelia
CRD: conformation-related disease
DCM: dilated cardiomyopathy
DT: Dogs Trust
EPRSC: Engineering and Physical Sciences Research Council
ESRC: Economic and Social Research Council
GADAG: Give A Dog A Genome
HAI: human-animal interactions
HBC: human behaviour change
KC: The Kennel Club
KCCT: Kennel Club Charitable Trust
MRC: Medical Research Council
MVD: mitral valve disease
NC3Rs: National Centre for the Replacement, Refinement and Reduction of Animals in Research
NERC: Natural Environment Research Council
PDSA: People’s Dispensary for Sick Animals
QCA: qualitative content analysis
RCVS: Royal College of Veterinary Surgeons
RVC: Royal Veterinary College
RVC ACT: Royal Veterinary College Animal Care Trust
RSPCA: Royal Society for the Prevention of Cruelty to Animals
SCAS: Society for Companion Animal Studies
UFAW: Universities Federation for Animal Welfare
UKRI: UK Research and Innovation
