# Supplementary material for: Researcher, research thyself? Mapping the landscape of canine health and welfare research funding provided by UK not-for-profit organisations from 2012–2022
Source: PLoS One. 2024 May 23;19(5):e0303498. doi: 10.1371/journal.pone.0303498 (PMC11115267; doi:10.1371/journal.pone.0303498)
Supplement: S1 Appendix — (DOCX) [file pone.0303498.s001.docx]

**Appendix 1: Big fish or small ponds: the significance of individual high-profile researchers in the canine health research sector**

This appendix uses in-scope canine-relevant research at the University of Surrey as a case study to demonstrate the influences that shape the detailed landscape of specialist canine health and welfare research. This veterinary school opened in 2014, and so is still establishing its expertise in the canine-relevant research sector. Surrey received the smallest amount of canine-relevant funding among all the established vet schools in the study (excluding those currently in the process of launching), with awarded canine-relevant grants totalling just over £911K (see Table 1): this probably reflects both its relatively recent launch and its disseminated practice-based clinical teaching model, with no centralised referral hospital. Moreover, one single grant accounted for just over half this money: a One Health multi-species study of zoonotic helminthiasis in the Philippines, funded by the MRC. The balance, £448K, was coded as canine-relevant funding.

**Appendix Table 1. Canine-relevant research at the University of Surrey, 2014-2022 (see main manuscript for list of funder abbreviations)**

| **Funder** | **Year** | **No. of grants** | **Amount** | **Subject** | **Benefit score (median if > 1 grant)** | **Pathway score (median if > 1 grant)** |
| --- | --- | --- | --- | --- | --- | --- |
| **Wide-scope** |  |  |  | **One Health** |  |  |
| MRC | 2018 | 1 | £463,333.88 | Multispecies study of zoonotic intestinal worms in Philippines | 15 | 8 |
| **Animal-directed** |  |  |  | **Canine-focused (not breed related)** |  |  |
| BSAVA PetSavers | 2021 | 2 | £19,694.14 | Clinical workplace technology studies | 5 | 6.5 |
| KCCT | 2022 | 1 | £27,889.00 | Owner symptom monitoring for cancer patients | 15 | 10 |
|  |  |  |  | **Canine-focused (breed related)** |  |  |
| KCCT | 2015 | 1 | £101,621.00 | Gait analysis in German Shepherd Dogs | 12 | 6 |
| Dogs Trust | 2014, 2020 | 2 | £206,995.00 | CM/SM genetics; CM/SM AI tool | 16 | 6.5 |
| Breed group (Cavalier Matters) | 2014 -2021 | at least 5 | £57,600.00 | Multiple phases of CM/SM research | 16 | 7 |
| BSAVA PetSavers | 2014-2018 | 3 | £5,047.35 | Small CM/SM projects | 16 | 6 |
| PetPlan Charitable Trust | 2016 | 1 | £10,000.00 | Machine learning and CM/SM | 12 | 8 |
| Waltham Foundation | 2017 | 1 | £19,170.00 | Machine learning and CM/SM | 16 | 7 |

Of this £448K, £47.6K was directed towards canine-focused clinical research that did not concern breed-related disease; this comprised two small BSAVA grants for workplace technology audit projects, and one KCCT grant to develop remote symptom monitoring technology to improve the quality of life for cancer patients. The remaining £400.4K was used to research breed-related disease. Apart from one KCCT-funded project, with a grant of £101.6K, which investigated locomotor problems in German Shepherd Dogs through gait analysis, this money (£298.8K) all supported research into Chiari-like malformation/syringomyelia (CM/SM) in the Cavalier King Charles Spaniel (CKCS) and other affected breeds, led by the veterinary neurologist Clare Rusbridge. This one research topic thus accounted for almost a third (32.8%) of Surrey’s total canine-relevant funding during the period of this analysis.

CM/SM is extremely common in predisposed breeds, particularly the CKCS, and for severely affected dogs can be devastating; the median ‘benefit for the dog’ (BFD) score (see main paper) for this research was 16, supporting the significance of this work to canine health. Professor Rusbridge is a leading researcher not only within companion animal science at Surrey, but in the global world of neurology, with a lengthy publication record. Moreover, this programme accounts for most CM/SM clinical research in the UK; this audit identified less than £10K of research at other institutions that was explicitly targeted at this problem. Yet, despite its obvious value, this research has only been possible through a series of grants from multiple animal-directed funding bodies and cross-subsidy from private clinical practice. Dogs Trust provided £207K (69%) of this funding, through two major grants. The second biggest source of funding, however, was Cavalier Matters, a breed-specific registered charity established and run by dog owners, which provided £57.6K of funding across multiple specific projects, contributing 19% of the total. Small grants from other animal-directed funders contributed the balance. This illustrates the piecemeal funding that canine-specific research, however valuable, must often rely on, reveals the potential significance of breed community activism in responding to breed-related disease problems, and shows how individual high-profile researchers can create centres of deep expertise that nevertheless are dependent on uncertain and precarious support.
